# Microscopy and chemical analyses reveal flavone-based woolly fibres extrude from micron-sized holes in glandular trichomes of *Dionysia tapetodes*

**DOI:** 10.1186/s12870-021-03010-9

**Published:** 2021-06-17

**Authors:** Matthieu Bourdon, Josephine Gaynord, Karin H. Müller, Gareth Evans, Simon Wallis, Paul Aston, David R. Spring, Raymond Wightman

**Affiliations:** 1 The Sainsbury Laboratory, University of Cambridge, Bateman Street, Cambridge, CB2 1LR UK; 2 Department of Chemistry, University of Cambridge, Lensfield Road, Cambridge, CB2 1EW UK; 3 Cambridge Advanced Imaging Centre, Department of Physiology, Development and Neuroscience, Downing Street, Cambridge, CB2 3DY UK; 4 Cambridge University Botanic Garden, 1 Brookside, Cambridge, CB2 1JE UK

**Keywords:** Cell wall, *Dionysia*, Farina, Flavone, Glandular trichome, Hydroxyflavone, Vacuole, Wool

## Abstract

**Background:**

*Dionysia tapetodes*, a small cushion-forming mountainous evergreen in the Primulaceae, possesses a vast surface-covering of long silky fibres forming the characteristic “woolly” farina. This contrasts with some related *Primula* which instead form a fine powder. Farina is formed by specialized cellular factories, a type of glandular trichome, but the precise composition of the fibres and how it exits the cell is poorly understood. Here, using a combination of cell biology (electron and light microscopy) and analytical chemical techniques, we present the principal chemical components of the wool and its mechanism of exit from the glandular trichome.

**Results:**

We show the woolly farina consists of micron-diameter fibres formed from a mixture of flavone and substituted flavone derivatives. This contrasts with the powdery farina, consisting almost entirely of flavone. The woolly farina in *D. tapetodes* is extruded through specific sites at the surface of the trichome’s glandular head cell, characterised by a small complete gap in the plasma membrane, cell wall and cuticle and forming a tight seal between the fibre and hole. The data is consistent with formation and thread elongation occurring from within the cell.

**Conclusions:**

Our results suggest the composition of the *D. tapetodes* farina dictates its formation as wool rather than powder, consistent with a model of thread integrity relying on intermolecular H-bonding. Glandular trichomes produce multiple wool fibres by concentrating and maintaining their extrusion at specific sites at the cell cortex of the head cell. As the wool is extensive across the plant, there may be associated selection pressures attributed to living at high altitudes.

**Supplementary Information:**

The online version contains supplementary material available at 10.1186/s12870-021-03010-9.

## Background

The genus *Dionysia* contains 55 species, found across central Asia. They are very closely related to *Primula* and some species have historically moved backwards and forwards between the two genera [1]. Under the Angiosperm Phylogeny Project, *Dionysia* has been subsumed into *Primula* (https://www.mobot.org). However, the current Missouri/Kew “Plant list”, a working list of all plant species, still recognises *Dionysia* as a genus in its own right (http://www.theplantlist.org). Species of *Dionysia* are dwarf shrubs or woody perennials forming loose to densely compact cushions with terminal inflorescences. They grow at high altitude in mountain regions usually on limestone cliffs although granite, sandstone and dolomitic sites have also been recorded and are found in shaded or semi shaded conditions. *Dionysia tapetodes* [2] is the most widely distributed *Dionysia* with a range from the Kopet Dagh through NE Iran (mountains of Khorasan Province) to the mountains of Afghanistan [3]. This wide range helps to explain the variation within the species. They form large, rather flat cushions with yellow flowers in the wild and in cultivation [4].

For some species of *Primula* and *Dionysia*, a mealy deposit termed farina (latin “meal” or “flour”) is found to cover all or a subset of aerial parts of the plant. It is readily observed, for example on the leaf surface, either as a powder or, in some species, as long fine thread (or wool)-like fibres that are commonly referred to as “woolly farina”. Woolly farina is common among *Dionysia* but comparatively rare in *Primula* [1]. For both genera, farinose and efarinose forms can exist within the same species and the underlying reasons for the latter variants are not well understood and may be unrelated. Powdery farina of *Primula* is mostly comprised of 2-phenyl-4H-chromen-4-one, more commonly known as flavone [5, 6]. Chemical analysis of solvent-rinsed plant organs reveal a growing number of additional substituted flavones, particularly hydroxy- and methoxy- derivatives. The number of different flavone types can be extensive and their presence/absence can vary between closely-related species [7–10]. Given these flavone assignments represent total surface-extracted flavones that include different tissue/cell types and the sticky resiniferous trichomes, it is not clear which substituted flavones actually constitute the farina of a given species.

Farina biosynthesis takes place in specialized glandular trichomes that typically consist of a stalk attached to a single round cell known as the glandular head [11–13]. The crystals of powdery farina are seen to coat the head cell and, at the subcellular level, a proliferation of smooth ER is observed suggesting a site of synthesis and transport of some biosynthetic intermediates [14, 15]. Part of the biosynthetic pathway to flavones is known but for synthesis of more complex derivatives is less well understood [16]. Chalcone synthase (CHS) represents the first step in the flavonoid biosynthetic pathway and immunolocalization of the enzyme in farinose *Primula kewensis* shows high signal in the gland head cell (but not in an *efarinose* mutant), suggesting the full flavone farina biosynthetic machinery is present in this cell [17]. Furthermore, immunogold labelling showed enrichment of CHS in spherical bodies in the cytoplasm. As flavone farina biosynthesis continues, the subcellular locations of intermediates and final products are not known. Generally, flavone glycosides, being soluble, can accumulate in the vacuole [18] but, for farina producing plants, the storage location of the insoluble aglycone is unknown. These flavone and flavone-type aglycones need to be transported out of the cell and through the cell wall and cuticle for deposition on the surface of the hair cell. Presumably this becomes more complicated for woolly farina whereby the flavone building blocks need to be concentrated to a single exit site to produce an elongating fibre.

We present here our results showing woolly farina composition and the subcellular organisation of the glandular trichome head cell that is the site of farina synthesis in *D. tapetodes*. We identify the wool as a mixture that includes flavone and hydroxyflavones that emerge from the head cell and is threaded through distinct gaps in the cell wall and cuticle. The mechanism of wool formation is discussed.

## Results

### Woolly farina fibres of *Dionysia tapetodes* have distinct surface grooves and differ in composition to powdery farina

**Fig. 1:**
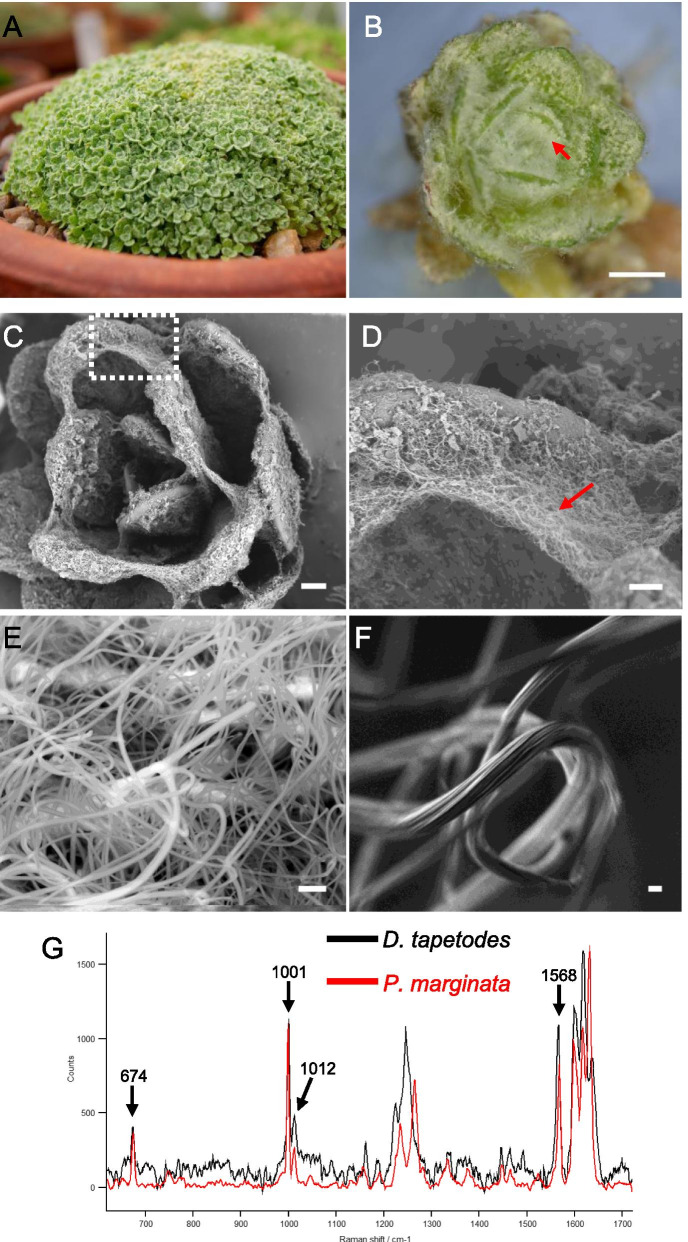
Farina wool observations on leaves of *Dionysia tapetodes.***a** Overview of the densely-packed, cushion-forming *D. tapetodes*. **b** Stereomicroscope image of white woolly farina (arrow) on leaves of *D. tapetodes.* Scale bar = 1 mm. **c** Scanning electron microscopy (SEM) of farina-coated leaves. Scale bar = 300 μm. Boxed region is magnified further in (**d**) where wool fibres can be observed between leaves (red arrow). Scale bar = 100 μm. **e** Detection of backscattered electrons by SEM showing array of wool fibres. Scale bar = 5 μm. **f** High magnification, low kV SEM image of a farina fibre showing the grooved surface structure. Scale bar = 1 μm. **g** Raman microscopy of fingerprint region of farina of *Dionysia tapetodes* (black) compared with that of the powdery farina from *Primula marginata* (red). Principle peak assignments (cm^−1^) common to both types of farina are indicated

*D. tapetodes* grows as a densely packed cushion (Fig. 1a) with large quantities of “woolly” fibres observed on both the adaxial and abaxial surface of the *D. tapetodes* leaf (Fig. 1b). Woolly farina production likely occurs throughout leaf growth since it is present in both young leaves (found at the centre of the rosette) and older leaves (positioned on the outside of the rosette). Farina production at early stages of growth are consistent with the observed entanglement of fibres between neighbouring leaves as revealed by scanning electron microscopy (SEM) (Fig. 1c, d). The wool itself comprises a meshwork of fibres (Fig. 1e). Individual woolly fibres range in width from 0.9 to 2.1 microns, with a mean width of 1.6 microns (*n* = 51). High magnification low kV SEM of uncoated fibres shows fine grooves on their surface, making a series of ridges arranged longitudinally (Fig. 1f). In order to understand how these fibres are formed and maintained, we compared the Raman spectra of woolly farina of *D. tapetodes* with the powdery farina found near the leaf margin of *Primula marginata*. Figure 1g shows an overlay of the spectra from both farina types. The powder spectrum of *P. marginata* matches that of pure flavone (see materials and methods for details of spectra correlation), indicating that flavone makes up all or the vast majority of the farina in this species. The spectrum of *D. tapetodes* woolly farina, however, only shares some of the peaks with the flavone powder of *P. marginata*; at 674, 1001, 1012 and 1568 cm^−1^. This suggests a substituted and/or mixture of flavones comprise the woolly farina of *D. tapetodes*. Spectral search and correlation software were used to identify candidate functional groups (see materials and methods) based on the acquired Raman spectra and compared to a reference database that included some flavone derivatives. While *P. marginata* farina correlated very highly with unsubstituted flavone (hit quality index 97 out of a possible 100 maximum), *D. tapetodes* woolly farina gave no close matches. However, the best hits (hit quality index 70–80) were to various combinations of mono-and di- hydroxy- and methoxy- substituted flavones. This suggests that hydroxy- and/or methoxy functional groups might co-exist with flavone in the wool fibres. Further evidence pointing to a correlation between mixed (substituted-)flavones and existence of fibres comes from Raman analysis of the atypical short fibres that surround the winter leaf trichomes of a Scottish isolate of *Primula bullata* var *bullata* (Additional file 1). The Raman spectrum of *P*. *bullata* farina is distinct from that of the flavone powder of *P. marginata* and is very similar (but not identical) to the spectrum of the long fibres from *D. tapetodes* (see spectral overlays in Additional file 1).

### The woolly farina of *D. tapetodes* comprises mostly flavone mixed with substituted flavones

**Fig. 2:**
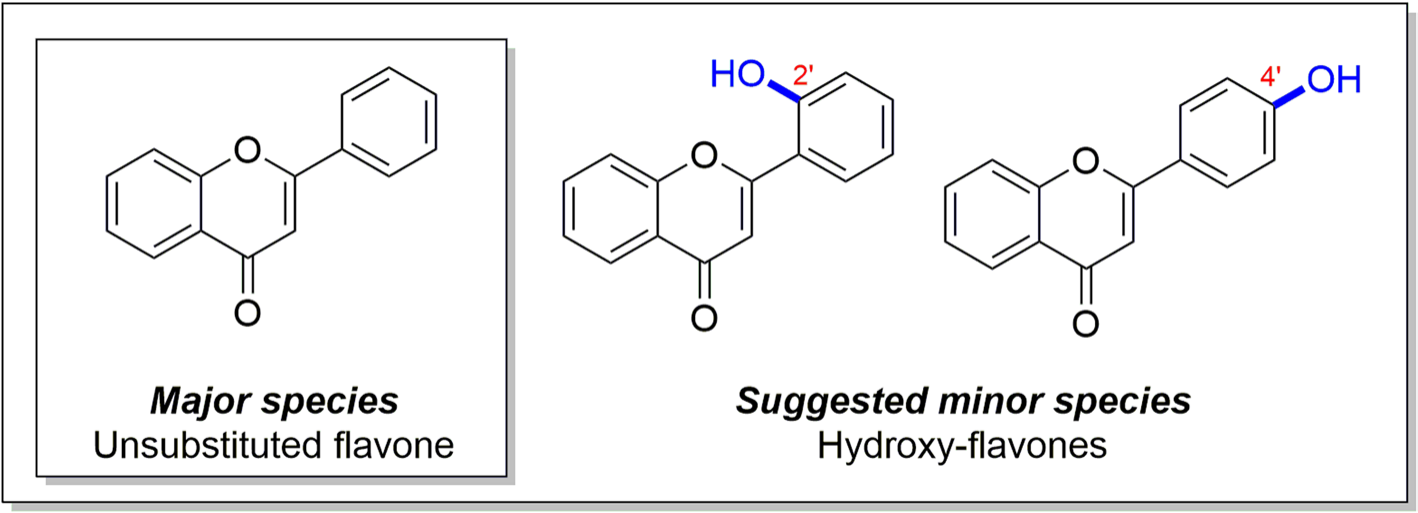
Molecular structures of candidate molecular species that form the woolly farina of *D. tapetodes*. Structures are derived from chemical analyses that are shown in full in Additional file 2

A sample of the woolly farina from *D. tapetodes* was further analysed by high-performance liquid chromatography (HPLC), liquid chromatography-mass spectrometry (LCMS), high-resolution mass spectrometry (HRMS) and nuclear magnetic resonance (NMR) spectroscopy. All data are presented together with a detailed interpretation in Additional file 2 that additionally comprises Figs. S1-S7 and Table S1. This analysis revealed that the woolly farina sample was mainly composed of unsubstituted flavone (> 90% by analytical HPLC, structure A in Fig. 2). Identical analytical data was obtained for a pure sample of flavone from a commercial source (Additional file 2, Fig. S4). The HPLC, LCMS and HRMS data indicated that there are other, substituted flavone species present in the woolly farina sample; exactly how many other species is not clear. HPLC analysis gave only one, whereas LCMS data suggested the presence of multiple other flavonoids. Mass ions were observed in the LCMS corresponding to hydroxy- and methoxy-substituted flavones (Additional file 2, Figs. S1 and S2). To provide more information on the structure of the minor flavone species, 2D NMR was carried out and yielded candidate structures corresponding to 2′-hydroxyflavone and 4′-hydroxyflavone (Fig. 2 and Additional file 2, Fig. S7).

### The wool fibres emerge from distinct holes in the cell wall of the glandular trichome head cell and are in close proximity to dense material of the vacuole

**Fig. 3:**
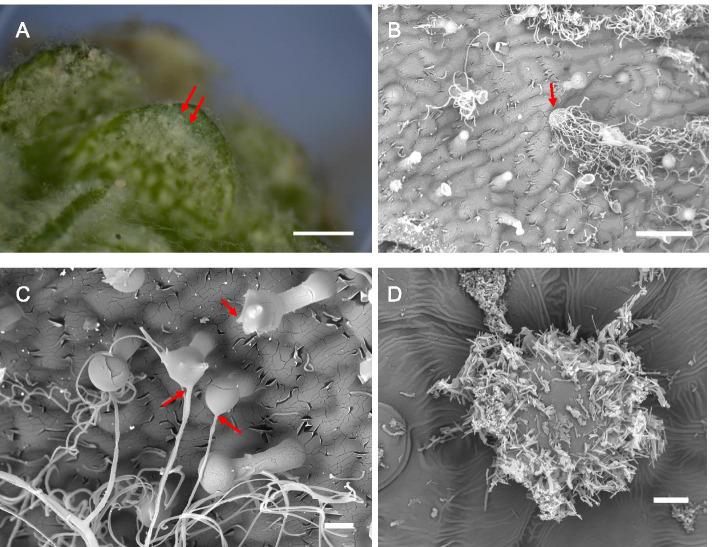
Formation of farina at the single cell level. **a** Stereomicroscope image showing examples of wool exit points (red arrows) on the surface of the leaf of *D. tapetodes*. Scale bar = 500 μm. **b** SEM image of glandular trichomes, including an example of a mature glandular trichome producing large quantities of woolly farina (red arrow). Scale bar = 50 μm. **c** SEM image showing farina wool exit points (red arrows) from glandular trichomes. Scale bar = 10 μm. **d** SEM image showing glandular trichome of *P. marginata* covered with powdery flavone farina. Scale bar = 10 μm

**Fig. 4:**
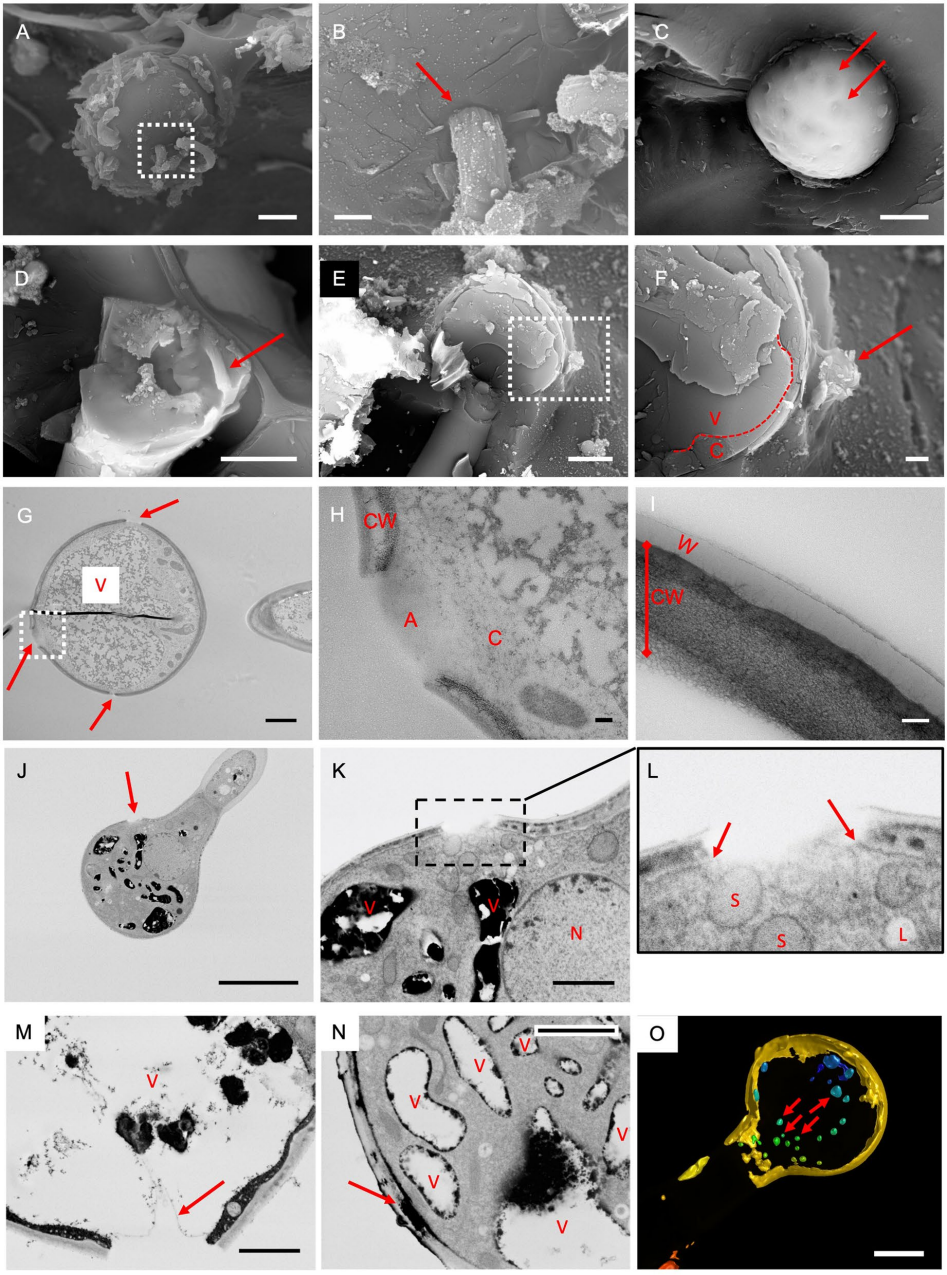
Exit points of farina wool from glandular trichomes. **a** SEM image showing wool exiting from the surface of a glandular trichome cell. A magnified view of the boxed region is shown in (**b**). Cell surface-farina interface is labeled by a red arrow. Bars = 5 μm (**a**) and 1 μm (**b**). **c** Surface of glandular trichome cell after ethanol wash to remove farinose material. Examples of wool exit points are labeled by red arrows. Bar = 5 μm. **d** SEM after cryo-fracture showing inner face of part of the surface of a trichome cell. A piece of farina wool is found to be present intact inside the cell (red arrow). Bar = 5 μm. **e** SEM after cryofracture revealing cell contents. Bar = 5 μm. A magnified view of the boxed area is shown in (**f**) where wool exit is observed (red arrow). The dashed line represents the location of the tonoplast. V = Vacuole, C = cytoplasm. The Vacuole is seen to be in close proximity to the wool exit site. Bar = 1 μm. **g** Transmission EM (TEM) image of section through glandular trichome. Wool exit sites are marked by red arrows and are defined as a gap in the cell wall. Electron dense material, consistent with vacuole contents [V], are in close proximity to these sites. Dark line in the centre is an artefact from a fold in the section. Bar = 2 μm. **h** a magnified view of a wool exit site (boxed region) showing a gap in the cell wall [CW] and an amorphous region (**a**) within this gap. Beneath the amorphous region is observed a small amount of cytoplasm (**c**). Bar = 200 nm. **i** TEM image of a section of intact cell wall [CW] overlayed by a waxy low density layer (W). Bar = 100 nm. **j**, **k**, **l** FE-SEM block face imaging of a glandular head showing wool exit site (arrow in **j**) Bar = 10 μm. A magnified view of the area covering the exits site is shown in (**k**) together with the electron dense vacuoles [V] and the Nucleus [N] Bar = 2 μm. Further magnification (**l**) shows the plasma membrane does not traverse the gap (arrows). Organelles fitting the description of spherosomes (S) and leucoplasts (L) can be seen below the gap. **m** FE-SEM block face imaging showing exit site that extends to the vacuole. Bar = 2 μm. **n** A build-up of electron dense material accumulates within a void in the cell wall (arrow). Vacuoles [V] containing similar electron dense material are found in close proximity. Bar = 2 μm. **o** Surface render of a 3D deconvolved confocal stack of a glandular head cell showing Nile red-positive stained compartments (examples indicated by arrows). Bar = 5 μm

Focal points representing the origin of numerous fibres can be seen on the *D. tapetodes* leaf surface (Fig. 3a) corresponding to mature flattened glandular trichomes (Fig. 3b). Interspersed between these locations are numerous smaller and extended trichomes that have fewer, single or no wool fibres emerging from the head cells and likely represent glandular trichomes at different levels of maturity and also non-wool forming resiniferous trichomes (Fig. 3b and c). It is evident that, when looking at the smaller trichomes, farina emerges from a few discrete locations on the glandular head cell that may grow in number as the cell ages. This appears to be in contrast to *P. marginata*, where smaller crystals completely cover the circumference of the head cell (Fig. 3d). These data show that, for *D. tapetodes*, flavone incorporation and extrusion needs to be sustained at the same site on the (head) cell surface. We used cryoSEM to observe, close-up, the junction between wool fibre and cell (Fig. 4a, b) where the fibre appears to come out of the cell wall. We generated random cryofractures through the leaf that might include a glandular head cell, however, the interaction of the fracture knives with the wool resulted in dislodging of the trichome from the leaf, as well as more cellular debris at the fracture site. We found a 5 s wash with 70% v/v ethanol removed the surface wool and resulted in better cryofractures with less debris. Sites of wool fibre extrusion, represented as small “craters” on the cell surface, are visible after the washing (Fig. 4c). Cryofractures that included any part of the gland head cell were, however, very rare. One fracture removed most of the head cell leaving a part of the head and some cytoplasm intact (Fig. 4d). Ethanol washing had removed the external wool fibres from the sample, however, a single fibre is observed inside the cell that was protected from the solvent (red arrow in Fig. 4d). This suggests the wool is made inside the cell and then somehow threaded through a gap in the wall. A second fracture was obtained through the centre of the gland hair cell (Fig. 4e) and a wool exit site could be observed close to the fracture plane. At this location the vacuole appeared to be close to the surface with a small amount of cytoplasm in between (Fig. 4f). Given that we could not obtain a fracture through the site of wool extrusion in the cell wall, we carried out Transmission Electron Microscopy (TEM) on post-stained sections of fixed glandular trichomes. TEM processing completely removes all the farina wool, however, complete gaps in the cell wall were observed (Fig. 4g) and were in close proximity to dense particulates that, based on the FE-SEM imaging described below, represent contents of the vacuole. These gaps are not an artefact from masking or non-uniform staining for TEM because they were also observed, in addition to the dense vacuoles, with light microscopy (Images are presented in Additional file 3). A small amount of cytoplasm, as well as an amorphous stained region, can be observed between the dense particulates and the cell wall gap (Fig. 4h). A magnified TEM view of the wall shows a mesh structure with layers of differing density that likely represent polysaccharide components including cellulose (Fig. 4i). A low density outer layer is present that is consistent with cuticular wax plus a thin epicuticular layer on top (Fig. 4i). This was confirmed by correlative fluorescence lifetime imaging and Raman microscopy which showed six Raman peaks (1062, 1132, 1294, 1416, 1438, 1465 cm^−1^) that correspond to the major peaks of epicuticular waxes from Sorghum [19]. These wax peaks were specific to the outer surface of the gland hair cell and were found together with the flavone-type signals (see lifetime-Raman data in Additional file 4). An intracellular store of flavone was also located.

In order to identify how the generation of a hole in the cell wall is coupled with directed deposition of flavone to make a thread, we used a higher throughput approach for imaging larger numbers of glandular trichomes. Full rosettes that include leaves at various stages of development were stained, embedded and sectioned. These large area sections were viewed by field emission scanning electron microscopy (FE-SEM) of backscattered electrons and gave good contrast for observing membranes. Out of 11 gaps examined, where vacuoles could be easily distinguished from other organelles due to their intense staining, 6 gaps were within 1 μm of its closest vacuolar compartment with distances in the range 0 – 2.6 μm (mean 1.1 μm, median 0.72 μm). FE-SEM showed the gaps in the cell wall and cuticle also includes the plasma membrane (Fig. 4j-l,); 15/15 gaps observed with FE-SEM had no plasma membrane crossing the gap. The lack of plasma membrane at these sites are unlikely due to membrane disruption due to fixation and processing as young developing trichomes and other leaf cells had intact membranes throughout (see images presented in Additional file 5). For one glandular head, we observed the gap to extend further inside the cell as far as the vacuole (Fig. 4m). We observed fully formed trichomes where the dense staining was seen in a void within the cell wall of width 1.15 μm consistent with the size expected of a forming wool thread (Fig. 4n) and suggesting a careful coupling between wall digestion and deposition of the flavone and derivatives. Although the close vicinity of the vacuole to the exit site suggests some role in thread formation, the typically hydrophilic environment of the vacuolar lumen is inconsistent with a role in storing flavone aglycones. A lipophilic compartment might be better suited as some final transport vehicle or store. In plants lipid/oil bodies or droplets have diverse morphologies and sizes, sometimes referred to as spherosomes for membrane bound organelles and leucoplasts for membrane-less protein-coated droplets [20]. Lipid-containing organelles are generally spherical or ovoid and either electron transparent or slightly opaque non-granular in EM prepared material [21, 22]. Putative spherosomes/leucoplasts can be seen below the cell wall gap in Fig. 4l but are not readily observed in this region in Fig. 4m. Comparing glandular heads with nearby leaf mesophyll cells does suggest these organelles are enriched in the latter cell types together with some electron dense droplets that are also visible (Additional file 6). Furthermore, confocal microscopy of trichomes stained with a lipophilic dye, Nile red, show evidence of intracellular positive stained compartments within the head cell (Fig. 4o, Additional file 7).

## Discussion

*D. tapetodes* is covered with woolly farina around the leaves. It is not entirely understood what the purpose of the farina is, however, tolerance to freezing and a block to high UV have been cited [23, 24]. Consistent with the latter, there exists an efarinose *D. tapetodes* accession at the Cambridge University Botanic Garden and horticulturists working closely with the plants report that it scorches more easily in the sun compared to the farinose accessions. Alternatively (or additionally) flavone-rich farina may have potent antifungal activity [25].

The woolly farina fibres radiate in all directions from mature glandular head cells of trichomes that are interspersed on the leaf epidermis. In addition to biosynthesis, these head cells appear to act as anchor points of the wool which can extend between adjacent leaves.

**Fig. 5:**
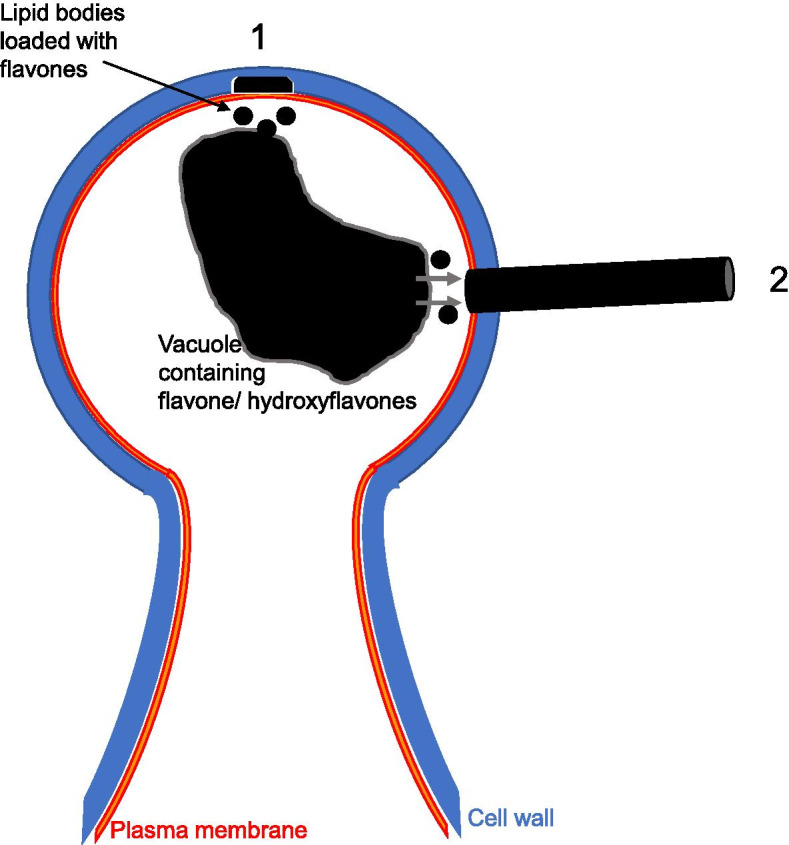
Proposed schematic of farina wool formation by the glandular trichome. (1) Localised cell wall digestion commences from the membrane face and is coupled with deposition of (hydroxy)flavones within the space. (2) Cell wall digestion produces a hole through which the (hydroxy)flavones are extruded. The (hydroxy)flavones may be deposited via lipid bodies or droplets and/or originating from glycoside precursors in the vacuole

The study of wool farina formation in *D. tapetodes* is made challenging by the physical and chemical characteristics of the wool itself. CryoSEM gave good preservation of wool and cellular structure but despite numerous random cryo-fractures, an image of a thread exiting the cell through the cell wall gap was never obtained. High resolution light and electron microscopy of embedded and sectioned material gave the best images of the wool exit sites and surrounding subcellular compartments, however, the processing removed the wool fibres (although dense vacuolar staining possibly attributed to stores of flavone precursors can be seen). Observing the gaps within the cuticle, cell wall, plasma membrane and cytoplasm where the thread is presumed to exist, before processing, does give us clues as to how the wool is made; we speculate that a small void in the wall, arising by digestion of the wall constituents, begins on the side of the wall facing the cell (Fig. 5). Flavone (aglycone) material loaded in to lipid bodies possibly from a vacuolar store (as a partially soluble glycone precursor) is transported to and deposited into this void. Completion of digestion to include the cuticle permits the flavone aggregate to then extrude outside the cell to form the wool while forming a perfect seal with the edge of the hole (Fig. 5), helped by the large amounts of epicuticular wax. For thread elongation, flavone is continually deposited to the cytoplasmic (elongating) end and the thread can protrude deeper into the cell with gradual extrusion to the outside possibly powered by cell turgor. It is conceivable that turgor-driven expulsion of the thread may result in vacuole displacement towards the exit site consistent with the observed close proximity with the vacuole.

A question remains as to how the woolly fibres of farina are formed and stably maintained where other flavone-based farina forms a powder. Our compositional analyses suggest the *D. tapetodes* wool is predominantly flavone mixed with 2′- and 4′-hydroxyflavones plus small quantities of other unidentified substituted flavones. The three identified flavones are not unique to this species, since they are found among powdery species, where they are referred to as epicuticular flavonoids [26]. There are, however, data supporting strong intermolecular H-bonding for the 2′ and 4′-hydroxy positions compared to weak H-bonds for the 6-hydroxy position and *intra*molecular bonds in 5-hydroxyflavone [27, 28]. These intermolecular interactions between the 2′ and 4′-hydroxyflavones and with the bulk flavone may be enough to maintain the integrity of a wool thread. Our Raman data of the *P. marginata* powder show a match with only flavone with no evidence of a mixture like that observed for *D. tapetodes* and so in the former species promoting powder rather than wool extrusion seen in the latter. Despite this observation, chemical analysis of exudate flavonoids demonstrate large quantities of flavone and 2′-hydroxyflavone in *P. marginata* [10]. We put forward a hypothesis that, while a diversity of substituted flavones is maintained across powdery and woolly farina forming species, only woolly farina species can correctly mix and incorporate these at a single site of synthesis to make and stabilise long continuous fibres. The precise differences in the mixtures that give rise to fine powder, coarse powder, short fibres or long fibres may be subtle yet sufficient for the big differences in farina morphology. The selection pressure(s) that resulted in powdery vs woolly vs efarinose is not understood, however, the wool does appear to give better coverage over the whole plant while we speculate that the fine loose powder, while more local and easier to dislodge, can yield a denser coating. In addition to the reported capacity of flavonoids to provide protection against UV and potentially during freezing events, the capacity of *D. tapetodes* for long-threading flavones may provide another evolutionary step towards previously described xeromorphic adaptation to drought in the genus *Dionysia* [29]. Indeed, such specific spatial deposition of flavones seems to offer more leaf coverage from a single production point (glandular trichome), which may limit air movement on the leaf surface, therefore limiting water loss.

## Conclusions

Woolly farina is formed by targeting a mixture of flavone and hydroxyflavones to exit holes within the glandular trichome head cell. We propose fibre integrity involves intermolecular H-bonding and, taken together, our data allow for further evaluation of these fibres as potential new biomaterials.

## Methods

### Plant propagation and harvesting

We used *Dionysia tapetodes* accession 20,140,435 that was received from the Royal Botanic Garden Edinburgh (accession 19,822,508) comprising wild material collected by Prof. T.F. Hewer (number 1164) between 1969 and 1971. *D. tapetodes* was grown at Cambridge University Botanic Garden (Cambridge, UK) in clay pots plunged in sand in an alpine house to keep the roots cooler and at a more stable temperature. The potting compost comprised 50% loam-based compost, 30% 1-9 mm grit, 10% sharp sand, 10% seramis and a small amount of slow release fertiliser. The compost is top-dressed with grit which is carefully worked under the collar of the plant to reduce the risks of basal rot. Given that *Dionysia* are prone to rosette burning from overexposure to sunlight, a temporary shading screen was placed over the collection from mid-March and removed later in the season after sun intensity decreased. Old flowers were carefully removed to prevent any botrytis infection moving from dead flowers into living material. Watering adhered to a strict regime: Young plants were watered from underneath via a water bath and once they passed one year old were then watered overhead without getting the cushion surface wet. Water was applied sparingly in the winter months and increased once the plants were in active growth. *Primula marginata* (accession 195,844,928) leaf samples were taken from plants grown in the mountain glasshouse at Cambridge University Botanic Garden. *Primula bullata* var *bullata* winter leaves were a gift from Prof. David Rankin (University of Edinburgh, UK).

### Cryo-Scanning Electron Microscopy (cryoSEM) and cryo-fracture

Rosettes of 5–8 leaves were mounted, frozen in nitrogen slush, platinum coated and fractured as previously described [30]. To accommodate better fractures without dislodging trichomes, some samples were dipped in 70% v/v ethanol to remove the wool and then air-dried prior to freezing. Cryo prepared samples were viewed using a Zeiss EVO HD SEM fitted with a backscattered electron detector and 25 kV acceleration voltage.

### SEM imaging of wool fibres

For high magnification, low kV imaging, clumps of uncoated wool fibres were placed on a sticky carbon tab and mounted on an SEM stub in the Zeiss EVO HD SEM at high vacuum and 1 kV accelerating voltage using SE detector fast scanning with frame averaging to prevent wool movement. Backscattered electron imaging of wool was carried out using the BSD detector and 25 kV accelerating voltage.

### Embedding, sectioning and imaging of trichomes by transmission electron microscopy (TEM) and light (fluorescence) microscopy

The tip of the leaf (or leaf buds) was removed to ensure efficient fixative penetration, and then immediately treated as described in [31] for fixation, dehydration, and embedding steps. For fluorescence microscopy, semi-thin Sects. (1 μm) of the whole leaf bud were then obtained with a Leica EM UC7 ultramicrotome using a Histo Jumbo 8 mm diamond knife (DiATOME) and laid on droplets of sterile water on (uncoated) glass microscopy slides. In order to avoid folds on sections during the water drying process, slides were dried on a hot plate set at 55 °C (Leica). Slides were finally mounted in a 1:1 solution of AF1 Citifluor antifadent with PBS, containing calcofluor as a cell wall counterstain, and imaged with a confocal microscope (Zeiss LSM700).

For TEM observations, 100 nm thin sections were obtained with a Leica EM UC7 ultramicrotome using an ultra 45° diamond knife (DiATOME) and deposited on 200-300 μm mesh formvar-coated nickel grids.

Samples were post-stained in Reynold’s lead citrate (3 min) plus uranyl acetate (3 min). Thin sections were viewed in a Tecnai G20 electron microscope at 200 keV, 20 μm objective aperture. Images were taken with an AMT camera controlled by DEBEN software.

### Field Emission Scanning Electron Microscopy (FE-SEM) of leaf sections

Whole rosettes were dissected with razor blades to remove leaf tips and subsequently processed as previously described in [32]. Following dissection, samples were immediately submerged in fixative (2% formaldehyde, 2% glutaraldehyde, 2.0 mM calcium chloride in 0.05 M sodium cacodylate buffer at pH 7.40) under vacuum overnight at room temperature. After washing 5 times in deionised water (DIW), samples were osmicated (1% OsO_4_, 1.5% K_3_Fe(CN)_6_ in 0.05 M sodium cacodylate buffer, pH7.4) for 3 days, 4 °C. Then samples were washed 5 × with DIW and treated with 0.1% (w/v) thiocarbohydrazide in DIW for 20 min in the dark at room temperature. After washing 5 × in DIW, osmication was repeated for 1 h at RT with 2% OsO_4_/DIW then 5 washing steps in DIW. Samples were then block-stained with uranyl acetate block stain (2% uranyl acetate in 0.05 M maleate buffer, pH 5.5) for 3 days, 4 °C. Samples underwent a further 5 washes in DIW and then dehydrated in a graded series of ethanol (50% > 70% > 95% > 100% > 100% dry), then 100% dry acetone and then 100% dry acetonitrile, 3 × in each for minimum 5 min. Samples underwent infiltration with a 50/50 mixture of 100% dry acetonitrile/Quetol resin, minus BDMA, overnight, followed by 3 days in 100% Quetol (minus BDMA) and then 5 days in 100% Quetol resin plus BDMA, exchanging the resin every 24 hr. The Quetol resin mixture consists 12 g Quetol 651, 5.7 g MNA, 15.7 g NSA, 0.5 g BDMA. Samples were placed within embedding moulds and then cured at 60 °C, 3 days. Semi-thin Sects. (1 μm) of the whole leaf bud were then obtained with a Leica EM UC7 ultramicrotome using a Histo-Jumbo 8 mm diamond knife (DiATOME) and then laid on droplets of sterile water on (uncoated) glass microscopy slides. Slides were dried on a hot plate at 55 °C (Leica) in order to avoid folds appearing during drying. Glass slides were then trimmed with a glass knifemaker to be mounted on aluminium SEM stubs using conductive carbon tabs, and the edges of the slides were painted with conductive silver paint. Samples were coated with 30 nm carbon using a Quorum Q150 TE carbon sputter coater. Samples were imaged in a FEI/Thermofisher Verios 460 SEM at 4 kV accelerating voltage, 0.2 nA probe current using a concentric backscatter detector (CBS) in immersion mode with a working distance of 3.5–4 mm; 1536 × 1024 pixel resolution, 3 μs dwell time, 4 line integrated. Stitching of adjacent image areas was carried out using the FEI MAPS software and default stitching settings and a 10% overlap.

### Measurements of wool fibre diameter

Images of wool fibres, attached to leaves of an isolated *D. tapetodes* rosette, were taken with a Keyence VHX-7000 microscope at 2500 × magnification and illuminated with full field coaxial light. 2D depth-up mode was used for in-focus acquisitions. Fibre width measurements were carried out using the point-to-point measuring tool in the Keyence software.

### Raman microscopy of farina

Raman microscopy was carried out on a Renishaw InVia instrument fitted with a 785 nm laser. *D. tapetodes* farina wool or *P. marginata* powder was carefully placed on a quartz slide and brought in to focus under a 50 × dry objective lens. Raman acquisitions used a 1200 l/mm grating, 1200 cm^−1^ centre, 785 nm laser at 10% power, regular confocal mode and 4 s exposure with 3 accumulations. At least 3 spectra per sample were averaged in order to improve signal-to-noise. To find close matches with reference Raman spectra, the experimental spectra were used as a search input against the Raman databases, that include some flavone derivatives, in the KnowItAll software (Bio-Rad Inc.) using the “SearchIT” tool and then candidate spectra were visualised by eye to remove false positives. Both default and Euclidean distance search settings were used. Matches are ranked according to their hit quality index (out of a maximum of 100). *P. marginata* farina gave a close match (97/100) with flavone. *D tapetodes* gave no close matches but yielded good correlation (70–80/100) to reference spectra of hydroxy- and methoxy- flavone derivatives that included 7, 2′-dimethoxyflavone; 3,7-dimethoxyflavone and 6-, 7-, or 8- hydroxy-derivatives. For fastFLIM-Raman correlative imaging of leaves submerged in water (Additional file 4) a confocal-Raman microscope, described in [33], used the following settings: 25 × 0.95 NA water dipping objective lens, FLIM 440 nm pulsed laser (at 20 MHz) with detector window set between 448 and 511 nm and 80 iterations. Raman: 1200 l/mm grating, 1200 cm^−1^ centre, 785 nm laser, 50% power, 15 s exposure with 2 accumulations used in line scan mode that intersected a glandular head cell.

### Nile red staining and imaging

Freshly harvested leaves of *D. tapetodes* were placed in tubes containing 0.1 μg/ml w/v of Nile red in 0.0001% acetone (prepared from a Nile red stock of 1 mg/ml w/v in 100% acetone). 3D Imaging was carried out on a Zeiss LSM700 confocal microscope using a 555 nm laser and 575–625 nm emission filter. Deconvolution and surface rendering of the Z-stack was carried out on Huygens software (Scientific Volume Imaging, Netherlands).

### Reagents, solvents and sample preparation for chemical analysis

Pure Flavone was purchased from Alfa Aesar as a white solid with 99% purity (CAS no. 525–82-6, catalogue no. A13627) and used without further purification. All solvents were anhydrous and used as purchased without any further purification. Flavone wool from was picked from *D. tapetodes* leaf surfaces using fine tweezers and placed in a microcentrifuge tube. The wool sample (approximately 0.5 mg) was dissolved in 50 μL of acetonitrile/water (1:1) with a few drops of dimethylsulfoxide (DMSO) to aid solubility. Sample preparation was performed in this way for analytical HPLC, LCMS and HRMS analysis.

### Analytical high-performance liquid chromatography (HPLC)

Analytical HPLC was run on an Agilent 1260 Infinity using a Supelcosil ABZ + PLUS column (150 mm × 4.6 mm, 3 μm) eluting with a linear gradient system (solvent A: 0.05% (v/v) trifluoroacetic acid (TFA) in H_2_O, solvent B: 0.05% (v/v) TFA in acetonitrile (MeCN)) over 15 min at a flow rate of 1 mL/min.

### Liquid chromatography mass spectrometry (LCMS)

Chromatographs were recorded on a Waters ACQUITY H-Class UPLC with an ESCi Multi-Mode ionisation Waters SQ Detector 2 spectrometer (LC system: solvent A: 2 mM ammonium acetate in water/MeCN (95:5); solvent B: 100% MeCN; column: AQUITY UPLC CSH C18, 2.1*50 mm, 1.7 μm, 130 Å; gradient: 63 5–95% B over 3 min with constant 0.1% formic acid).

### High resolution mass spectrometry (HRMS)

HRMS was carried out on a Waters LCT Premier Time of Flight mass spectrometer. ESI refers to the electrospray ionisation technique.

### Nuclear magnetic resonance (NMR) spectroscopy

NMR spectroscopy was carried out as described in reference [34], with the following modifications: All pulse sequences are the default (with the exception of the DEPT135) from the Topspin 3.2pl7 software used to control the acquisition. The analysis required ^1^H, ^13^C, DEPT135, DFQ-COSY, Heteronuclear Single Quantum Coherence (HSQC, with DEPT 135 editing) and Heteronuclear Multiple Bond Correlation Spectroscopy (HMBC) spectra. All necessary shaped and decoupling pulses were calculated by the software, using defined 90 degree pulses.

#### ^1^H NMR

Proton magnetic resonance spectra were recorded using an internal deuterium lock (at 298 K unless stated otherwise) on Bruker DPX (400 MHz; ^1^H-^13^C DUL probe), Bruker Avance III HD (400 MHz; Smart probe), Bruker Avance III HD (500 MHz; Smart probe) and Bruker Avance III HD 62 (500 MHz; DCH Cryoprobe) spectrometers. Pulse sequence used zg30 – PLW1 = 14 W, P1 = 10.5 μs, SW = 20 ppm, TD = 64 K, AQ = 3.28 s, D1 = 1 s, NS = 16. Proton assignments are supported by ^1^H-^1^H COSY, ^1^H-^13^C HSQC or ^1^H-^13^C HMBC spectra, or by analogy. Chemical shifts (δH) are quoted in ppm to the nearest 0.01 ppm and are referenced to the residual non-deuterated solvent peak. Discernible coupling constants for mutually coupled protons are reported as measured values in Hertz, rounded to the nearest 0.1 Hz. Data are reported as: chemical shift, multiplicity (br, broad; s, singlet; d, doublet; t, triplet; q, quartet; m, multiplet; or a combination thereof), number of nuclei, coupling constants and assignment.

#### ^13^C NMR

Carbon magnetic resonance spectra were recorded using an internal deuterium lock (at 298 K unless stated otherwise) on Bruker DPX (101 MHz), Bruker Avance III HD (101 MHz) and Bruker Avance III HD (126 MHz) spectrometers with broadband proton decoupling. 1024 scans (NS) were acquired using pulse sequence 'zgpg30′, with waltz16 1H decoupling. 90 degree 13C pulse set to 21 W (PLW1) for 9.5 μs (P1). 209,786 points (TD) were digitised over 3.02 s (AQ), relaxation delay set to 2 s (D1). Sweep width was 276 ppm (SW), with an irradiation frequency of 110 PPM (O1P). Carbon spectra assignments are supported by DEPT editing, ^1^H-^13^C HSQC or ^1^H-^13^C HMBC spectra, or by analogy. Chemical shifts (δC) are quoted in ppm to the nearest 0.1 ppm and are referenced to the deuterated solvent peak. Data are reported as: chemical shift, number of nuclei, multiplicity, coupling constants and assignment. Magnetic resonance spectra were processed using TopSpin (Bruker). An aryl, quaternary, or two or more possible assignments were given when signals could not be distinguished by any means. Standard flavone numbering was followed.

#### DEPT 135

Pulse sequence dept135sp – This is a minor modification of the DEPT sequence to optimise the spectral baseline and uses an adiabatic shape for 180 degree carbon pulses. Carbon pulse powers as ^13^C experiment above, SW = 236.7 ppm, TD = 65,536, AQ = 1.10 s, D1 = 2 s, O1 = 100 ppm, NS = 64. Waltz16 decoupling.

#### DFQCOSY

This is a double-quantum filtered experiment; using gradient selection; pulse sequence cosygpmfqf. Non-uniform sampling; using a Poisson-gap weighted schedule was used to acquire 37.5% of 512 increments, each with 2 scans (SWF2 = 13.37 ppm, TD = 4 k, AQ = 0.31 s, D1 = 2 s). Proton pulse powers as above. Processed to 2 k x2k points using a sine function (SSB = 2.5).

#### HMBC

This experiment is phase sensitive; uses Echo/Antiecho gradient selection, with a three-fold low-pass J-filter to suppress one-bond correlations; pulse program 'hmbcetgpl3nd'. Acquired in phase sensitive mode using Echo/Antiecho-TPPI gradient selection, with a 3 step low pass j-filter to suppress 1 bond correlations. Long-range J-JCH parameters set to 10 Hz. Non-uniform sampling; with a Poisson–gap weighted schedule was used to acquire 37.5% of 768 increments; each with 2 scans (SWF1 = 250, SWF2 = 12.02 ppm, TD = 4096, AQ = 0.34 s, D1 = 2 s). Processed to 2048 × 2048 using a sine function (SSB = 4 & 2 for F2 and F1), then converted to magnitude mode in F2. (Topspin command 'xf2m').

#### HSQC

The HSQC was acquired using a Bruker Avance III HD 500Mhz equipped with a dual 13C/1H cryoprobe; using Topspin 3.2pl7. It was acquired in 'non-uniform sampling' mode and samples 25% of 1024 increments, using a 'poisson-gap' schedule. The data was processed using the default compressed sensing (CS) method in Topspin 3.5pl7 (on your computer) to 2048 × 2048 data points. This experiment is configured to give -CH2 groups an opposite phase to the -CH and -CH3 groups, using the hsqcedetgpsp.3 pulse sequence. Acquired in phase sensitive mode using Echo/Antiecho-TPPI gradient selection, multiplicity edited during selection step with shaped adiabatic pulses. Non-uniform sampling; with a Poisson-gap weighted schedule was used to acquire 25% of 1024 increments; each with 2 scans (SWF1 = 190 ppm, SWF2 = 12.99 ppm, TD = 1816, AQ = 0.14 s, D1 = 0.8 s). Carbon and proton pulse powers as above. Processed to 2048 × 2028 points using a qsine function (SSB = 2).

Standard flavone numbering:

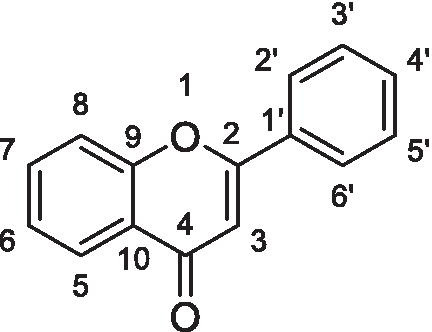



## Supplementary Information

**Additional file 1.** The farina of Primula bullata var bullata comprises very short fibres. SEM microscope images of the leaf farina threads is shown in (A). Red arrows indicate short threads. Wool thread diameter mean value and range is shown in (B). Raman spectra comparisons between P. bullata, Dionysia tapetodes and Primula marginata are shown in (C). Arrows show peaks that are shared between samples.

**Additional file 2.** All data and interpretations plus commentary for HLPC, LCMS, HRMS and NMR chemical analyses.

**Additional file 3.** Confocal transmitted image (A, C) and cell wall fluorescence (B, D) of calcofluor-stained sections through gland hair cells. Wool exit holes, observed as discrete gaps in the fluorescence images (arrows) are in close proximity to the dense vacuole (V).

**Additional file 4.** Fluorescence Lifetime Imaging (FLIM) of glandular trichomes taken together with Raman spectra acquired along a line through a trichome cell. The FLIM data (centre left image) represents lifetimes of autofluorescence. Farinose material including the wool and edge of the trichome cell have short lifetimes (cyan and blue colours) with high signal and the cell interior has blue (short) and green (long) lifetimes with low signal. Raman measurements at precise locations along the line confirm the presence of flavone-type material at the cell edge together with strong peaks equivalent to those of plant epicuticular wax (Upper spectrum, assignments are given for prominent peaks). Note the intense cyan labelling in the FLIM image that is a similar lifetime to the woolly farina (white boxed region). Within a proximal location inside the cell there is an absence of the wax-associated peaks and the strong flavone peaks (lower spectrum). Carotenoid is detected at both locations. Another location inside the cell yielded strong flavone peaks (centre spectrum) and may represent an intracellular store of flavones.

**Additional file 5.** FE-SEM images showing intact and continuous membranes from young trichomes and leaf cells. Note these images were taken from the same section as the farina producing glandular trichomes shown in Fig. 4j-n.

**Additional file 6.** FE-SEM large area tile-scan of a section thorough the rosette leaves of D. tapetodes. The high resolution tile images enable organelles to be resolved. Magnified examples of glandular head cells and nearby leaf mesophyll cells are shown. Red arrows identify candidate electron-transparent organelles that may be lipid droplets. Yellow arrows identify similar sized electron dense droplets. The full resolution tiled image of size 35,172 x 46,156 pixels is deposited at http://dx.doi.org/10.17632/tk534bkb85.1.

**Additional file 7.** Movie progressing through a single deconvolved confocal Z-stack of a D. tapetodes glandular trichome after treatment with the lipophilic dye, Nile red.

## Data Availability

The FE-SEM large area tile-scan of *D. tapetodes* leaves and the digital microscope measurements of wool fibres have been deposited at https://dx.doi.org/10.17632/tk534bkb85.1. Other datasets used and/or analysed during the current study are available from the corresponding author on reasonable request. Plant material requests should be directed to Cambridge University Botanic Garden.

## Abbreviations

BDMA: Benzyldimethylamine
BSD: BackScatter electron Detector
CBD: Concentric BackScatter Detector
CS: Compressed Sensing
DEPT: Distortionless Enhancement by Polarization Transfer
DFQCOSY: Double Filtered (Quantum) COrrelated SpectroscopY
DIW: Deionised water
DMSO: Dimethylsulfoxide
EM: Electron Microscopy
ESI: ElectroSpray Ionisation
FE-SEM: Field Emission Scanning Electron Microscopy
FLIM: Fluorescence Lifetime Imaging
HMBC: Heteronuclear Multiple Bond Correlation
HPLC: High Performance Liquid Chromatography
HRMS: High Resolution Mass Spectrometry
JCH: J-coupling constant for C-H bond
LCMS: Liquid Chromatography Mass Spectrometry
MW: Molecular Weight
NMR: Nuclear Magnetic Resonance
PBS: Phosphate-Buffered Saline
PPM: Parts Per Million
SE: Secondary Electron
SEM: Scanning Electron Microscope/Microscopy
TEM: Transmission Electron Microscopy
TFA: Trifluoroacetic acid
TPPI: Time Proportional Phase Incrementation
UV: Ultra Violet

## References

[1] Lidén M (2007). The genus Dionysia (Primulaceae), a synopsis and five new species. Willdenowia.

[2] Die BA, der Gattung A, Fenzl D (1871). Bull l’Académie impériale des Sci St Pétersbg.

[3] Grey-Wilson C. The genus Dionysia. Alpine Garden Society; 1989

[4] Beckett KA, Grey-Wilson C (1993). Alpine Garden Society (Great Britain).

[5] Müller H (1915). XCVI.—The occurrence of flavone as the farina of the primula. J Chem Soc Trans.

[6] Blasdale WC (1945). The composition of the solid secretion produced by primula denticulata. J Am Chem Soc.

[7] Hinterdobler W, Valant-Vetschera KM, Brecker L (2017). New primula -type flavones from exudates of selected dionysia spp. (Primulaceae). Nat Prod Commun.

[8] Bhutia TD, Valant-Vetschera KM, Brecker L (2013). Orphan flavonoids and dihydrochalcones from primula exudates. Nat Prod Commun.

[9] Valant-Vetschera KM, Bhutia TD, Wollenweber E (2010). Chemodiversity of exudate flavonoids in Dionysia (Primulaceae): A comparative study. Phytochemistry.

[10] Valant-Vetschera KM, Bhutia TD, Wollenweber E (2009). Exudate flavonoids of primula Spp: structural and biogenetic chemodiversity. Nat Prod Commun.

[11] Vitalini S, Flamini G, Valaguzza A, Rodond G, Iriti M, Fico G (2011). Primula spectabilis Tratt. aerial parts: morphology, volatile compounds and flavonoids. Phytochemistry.

[12] Bhutia TD, Valant-Vetschera KM (2012). Diversification of exudate flavonoid profiles in further primula spp. Nat Prod Commun.

[13] Fico G, Rodondi G, Flamini G, Passarella D, Tomé F (2007). Comparative phytochemical and morphological analyses of three Italian Primula species. Phytochemistry.

[14] Gunning BES, Steer MW. Plant cell biology: structure and function. Jones and Bartlett Publishers; 1996

[15] Wollenweber E, Schnepf E (1970). Vergleichende untersuchungen über die flavonoiden Exkrete von “Mehl”- und “Öl” -Drüsen bei Primeln und die Feinstruktur der Drüsenzellen. Z Pflanzenphysiol.

[16] Jiang N, Doseff AI, Grotewold E (2016). Flavones: from biosynthesis to health benefits. Plants (Basel, Switzerland).

[17] Schopker H, Kneisel M, Beerhues L, Robenek H, Wiermann R (1995). Phenylalanine ammonia-lyase and chalcone synthase in glands of Primula kewensis (W. Wats): immunofluorescence and immunogold localization. Planta.

[18] Marinova K, Kleinschmidt K, Weissenböck G, Klein M (2007). Flavonoid biosynthesis in barley primary leaves requires the presence of the vacuole and controls the activity of vacuolar flavonoid transport. Plant Physiol.

[19] Farber C, Li J, Hager E, Chemelewski R, Mullet J, Rogachev AY (2019). Complementarity of raman and infrared spectroscopy for structural characterization of plant epicuticular waxes. ACS Omega.

[20] Lersten NR, Czlapinski AR, Curtis JD, Freckmann R, Horner HT (2006). Oil bodies in leaf mesophyll cells of angiosperms: overview and a selected survey. Am J Bot.

[21] Freyre CAC, Rauher PC, Ejsing CS, Klemm RW (2019). MIGA2 links mitochondria, the ER, and lipid droplets and promotes De Novo lipogenesis in adipocytes. Mol Cell.

[22] Huang AHC (2018). Plant lipid droplets and their associated proteins: potential for rapid advances. Plant Physiol.

[23] Sisa M, Bonnet SL, Ferreira D, Van der Westhuizen JH (2010). Photochemistry of flavonoids. Molecules.

[24] Caldwell MM (1971). Solar UV irradiation and the growth and development of higher plants. Photophysiol Curr Top.

[25] Weidenbörner M, Jha HC (1997). Antifungal spectrum of flavone and flavanone tested against 34 different fungi. Mycol Res.

[26] Colombo PS, Flamini G, Christodoulou MS, Rodondi G, Vitalini S, Passarella D (2014). Farinose alpine primula species: phytochemical and morphological investigations. Phytochemistry.

[27] Looker JH, Hanneman WW, Kagal SA, Dappen JI, Edman JR (1966). Physical and chemical properties of hydroxyflavones. IV. Infrared absorption spectra of dihydroxyflavones containing the 5-hydroxyl group. J Heterocycl Chem.

[28] Looker JH, Hanneman WW (1962). Physical and chemical properties of hydroxyflavones. I. Infrared absorption spectra of monohydroxyflavones and their O-Methyl and O-Acetyl Derivatives 1,2. J Org Chem.

[29] Wendelbo P (1971). On xeromorphic adaptations in the genus Dionysia (Primulaceae). Ann des Naturhistorischen Museum Wien.

[30] Wightman R, Wallis S, Aston P (2018). Leaf margin organisation and the existence of vaterite-producing hydathodes in the alpine plant Saxifraga scardica. Flora.

[31] Alonso-Serra J, Shi X, Peaucelle A, Rastas P, Bourdon M, Immanen J (2020). ELIMÄKI locus is required for vertical proprioceptive response in birch trees. Curr Biol.

[32] Kunz D, Wang A, Chan CU, Pritchard RH, Wang W, Gallo F (2021). Downregulation of extraembryonic tension controls body axis Formation in avian embryos. bioRxiv.

[33] Wightman R, Busse-Wicher M, Dupree P (2019). Correlative FLIM-confocal-Raman mapping applied to plant lignin composition and autofluorescence. Micron.

[34] King TA, Stewart HL, Mortensen KT, North AJP, Sore HF, Spring DR (2019). Cycloaddition strategies for the synthesis of diverse heterocyclic spirocycles for fragment-based drug discovery. European J Org Chem.

