# Supplementary material for: Microscopy and chemical analyses reveal flavone-based woolly fibres extrude from micron-sized holes in glandular trichomes of Dionysia tapetodes
Source: BMC Plant Biol. 2021 Jun 17;21:258. doi: 10.1186/s12870-021-03010-9 (PMC8210372; doi:10.1186/s12870-021-03010-9)
Supplement: Supplementary file 1 — Additional file 1. The farina of Primula bullata var bullata comprises very short fibres. SEM microscope images of the leaf farina threads is shown in (A). Red arrows indicate short threads. Wool thread diameter mean value and range is shown in (B). Raman spectra comparisons between P. bullata, Dionysia tapetodes and Primula marginata are shown in (C). Arrows show peaks that are shared between samples. [file 12870_2021_3010_MOESM1_ESM.pdf]

A

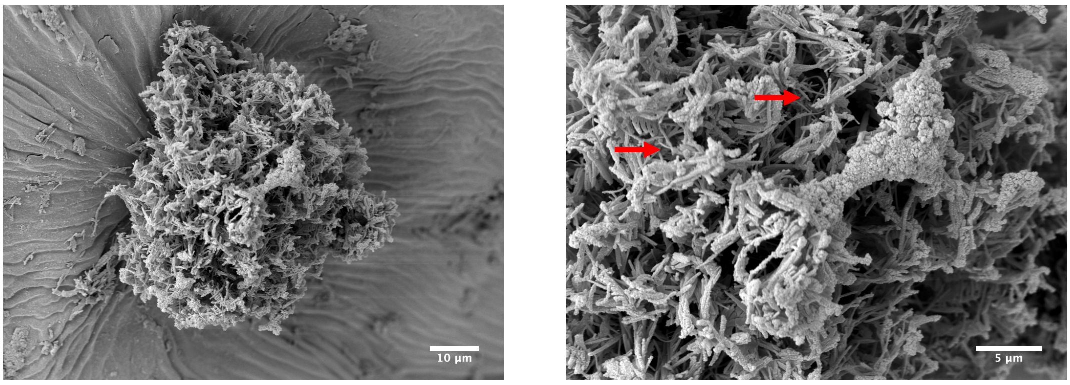

B

P. bullata wool diameter: Mean = 1.14 microns. Lower limit 0.68 microns. Upper limit 1.68 microns. n = 52.

C

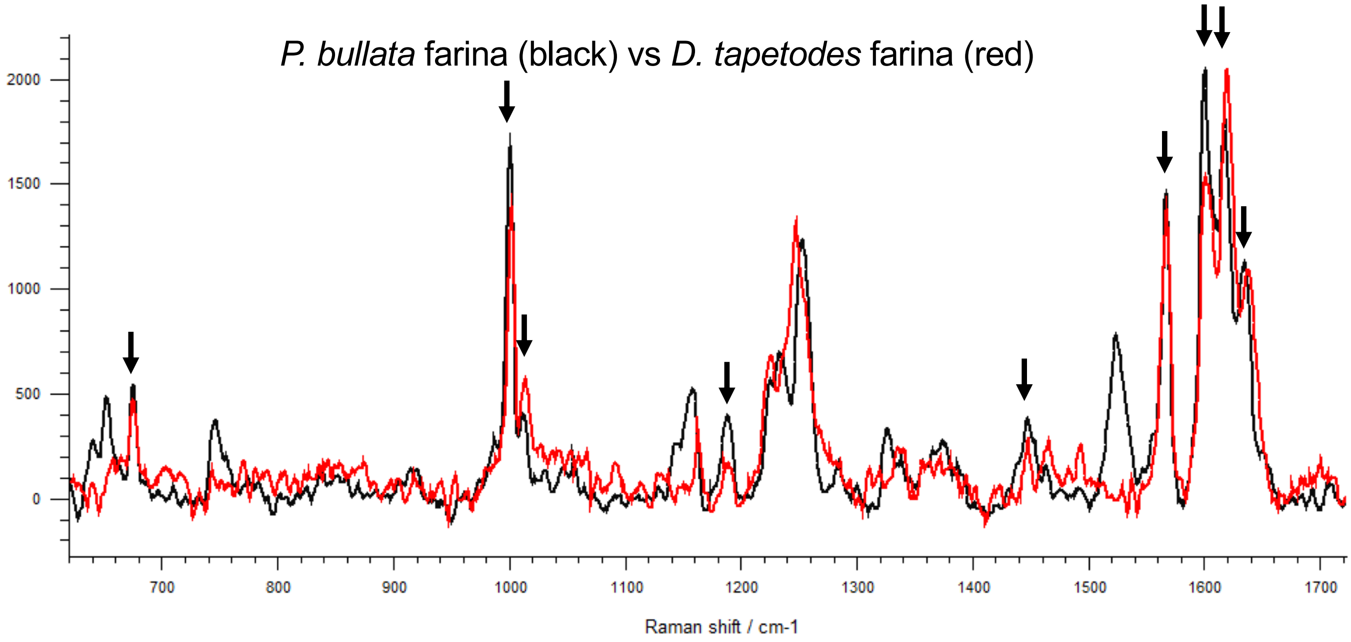

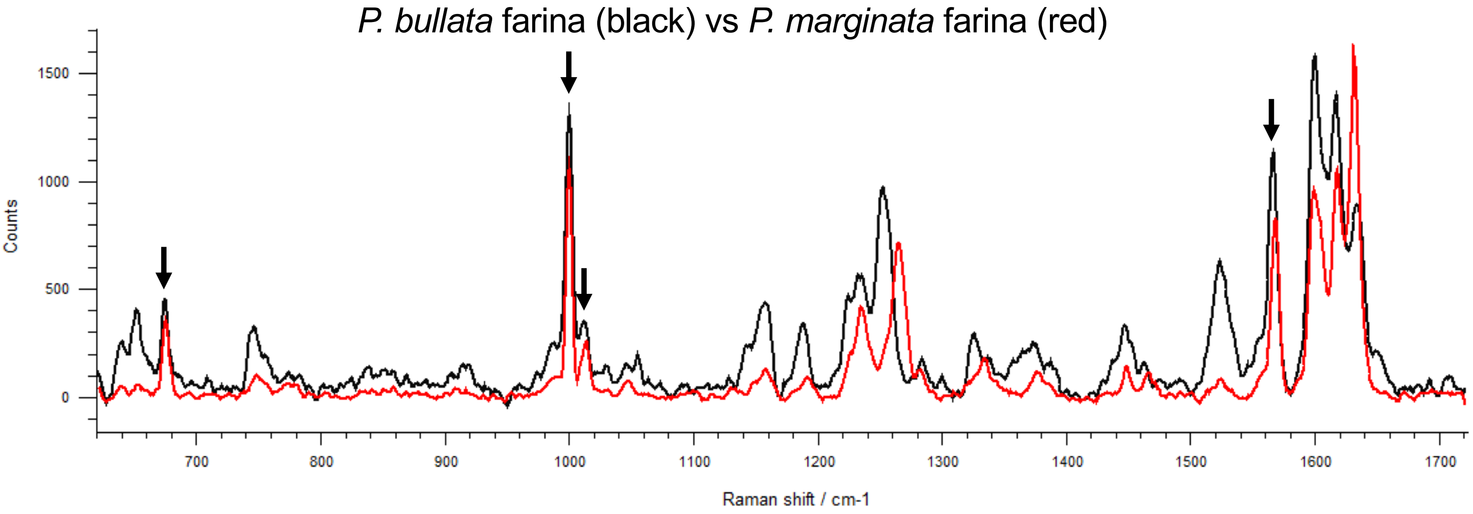

Additional file 2. The farina of *Primula bullata* var *bullata* comprises very short fibres. SEM microscope images of the leaf farina threads is shown in (A). Red arrows indicate short threads. Wool thread diameter mean value and range is shown in (B). Raman spectra comparisons between *P. bullata*, *Dionysia tapetodes* and *Primula marginata* are shown in (C). Arrows show peaks that are shared between samples.
