# Supplementary material for: Microscopy and chemical analyses reveal flavone-based woolly fibres extrude from micron-sized holes in glandular trichomes of Dionysia tapetodes
Source: BMC Plant Biol. 2021 Jun 17;21:258. doi: 10.1186/s12870-021-03010-9 (PMC8210372; doi:10.1186/s12870-021-03010-9)
Supplement: Supplementary file 2 — Additional file 2. All data and interpretations plus commentary for HLPC, LCMS, HRMS and NMR chemical analyses. [file 12870_2021_3010_MOESM2_ESM.docx]

**Analytical HPLC analysis of wool fibre samples**

To assess the purity of the sample, HPLC analysis was performed using two different gradients (5-95% and 40-95% acetonitrile in water) to ensure that all peaks present were observed.


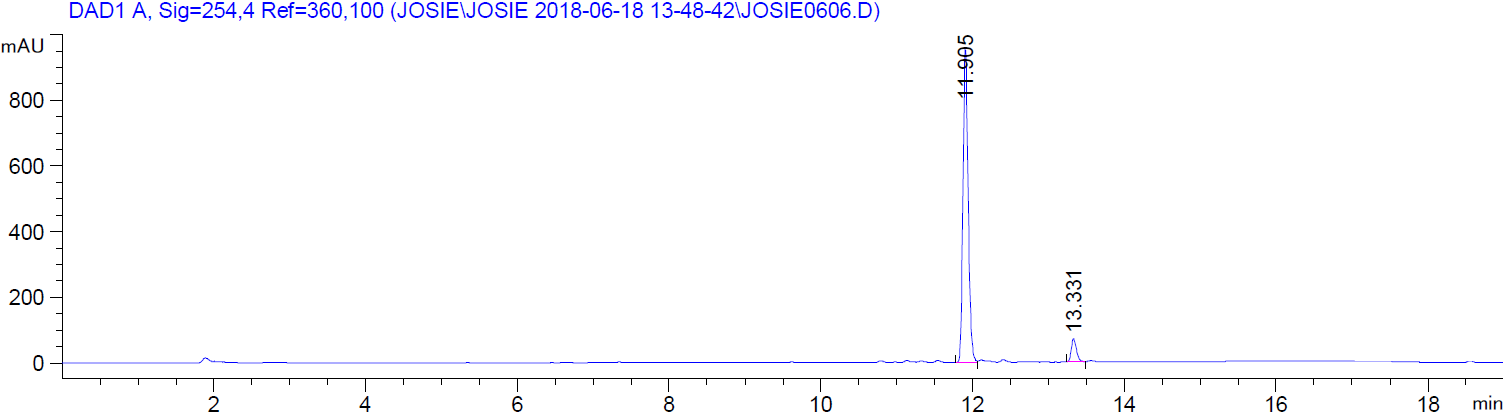


**Figure S1a** The HPLC spectra for analysis of the sample using a 5-95% acetonitrile in water gradient. The large signal at 2-2.5 minutes in the 220 nm trace (Figure 1 and 2) is due to dimethylsulfoxide (DMSO) which was used to dissolve the sample.


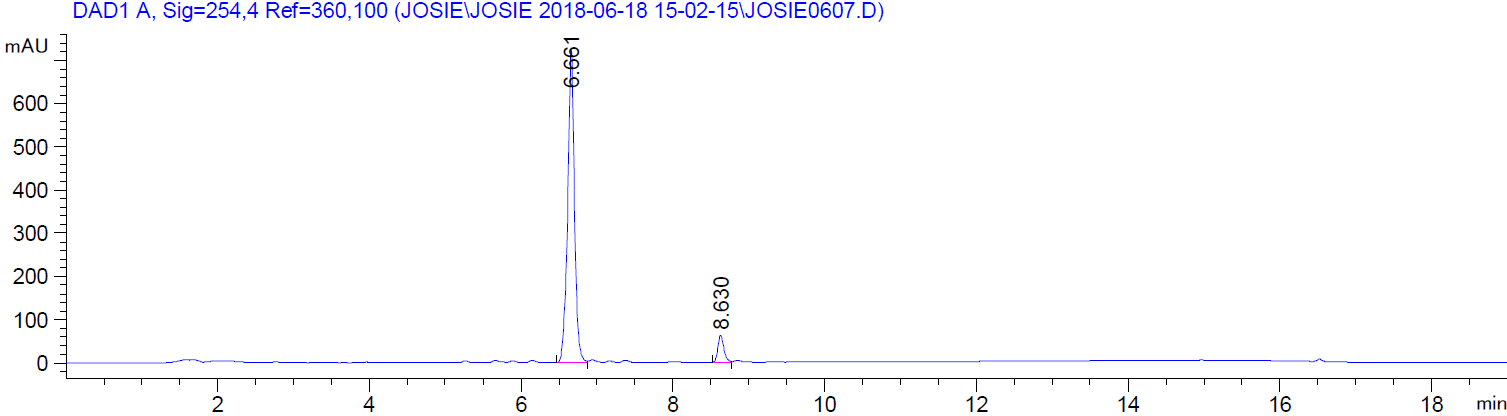


**Figure S1b** The HPLC spectra for analysis of the sample using a 5-95% acetonitrile in water gradient.

**Table S1** The peaks observed in the spectra shown in Figure 1.

| **Peak** | **Retention time (min)** | **Area (mAU*s)** | **Area (%)** |
| --- | --- | --- | --- |
| 1 | 11.905 | 4507.078 | 93.0 |
| 2 | 13.331 | 340.296 | 7.0 |
| 1 | 6.661 | 4535.346 | 93.0 |
| 2 | 8.630 | 339.714 | 7.0 |

**S2: Mass spectrometry of wool fibre sample**

**A**


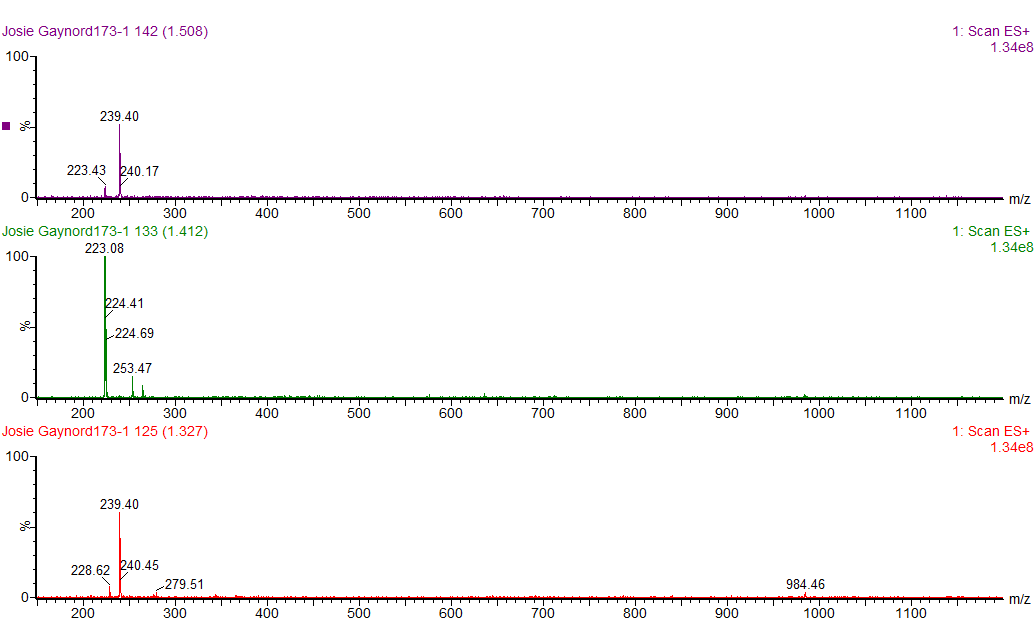

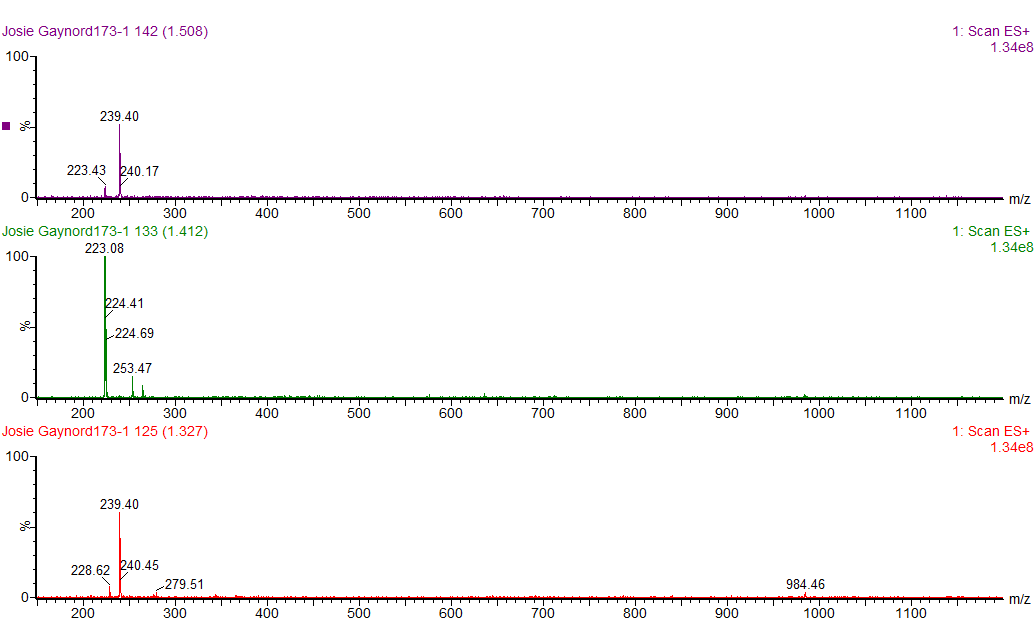

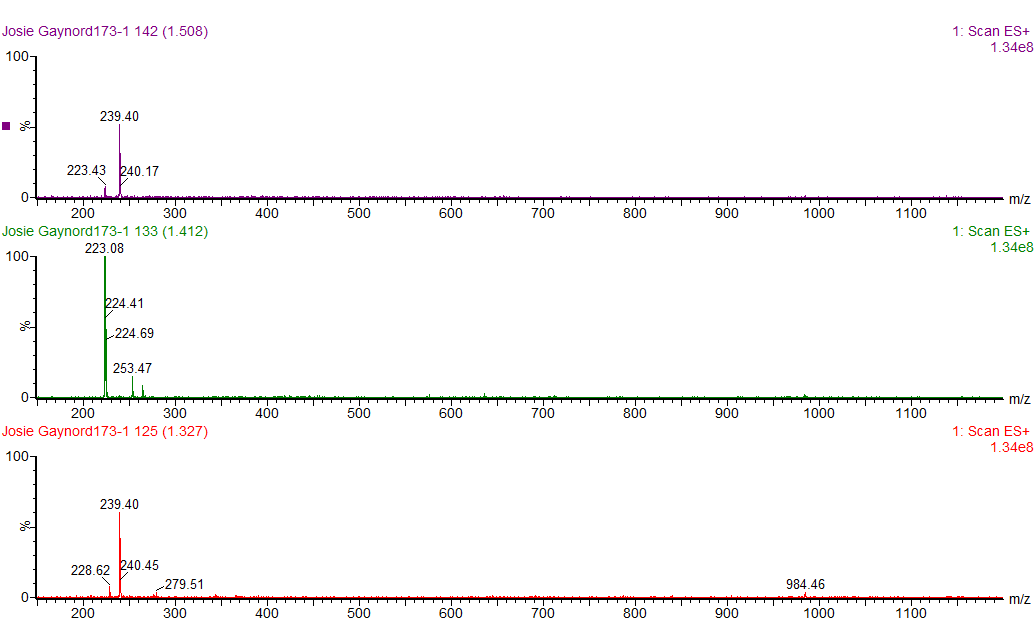

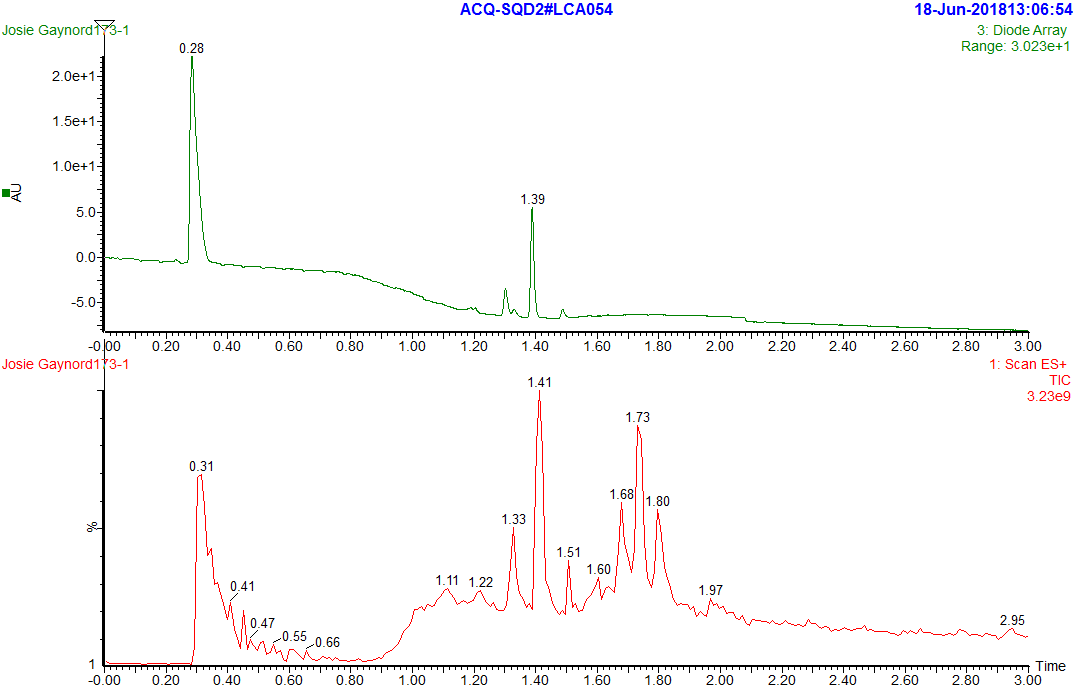


**B**

**Figure S2a** A: The UV trace for the LCMS spectrum of the plant sample. B: The corresponding ES+ trace. Insets are mass traces (ES+) for individual peaks, as indicated by the arrows. Suggested chemical structures with exact masses and molecular weights are shown over the relevant mass spectra.


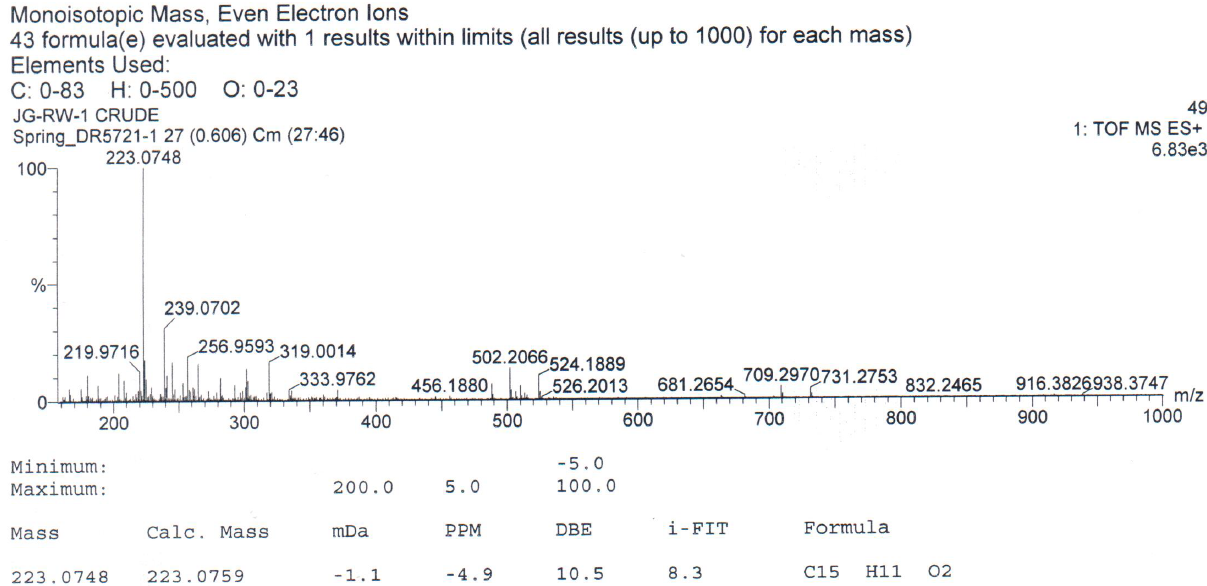


**Figure S3** The high-resolution mass spectrum (HRMS) for the plant sample.

**HPLC analysis of pure flavone**


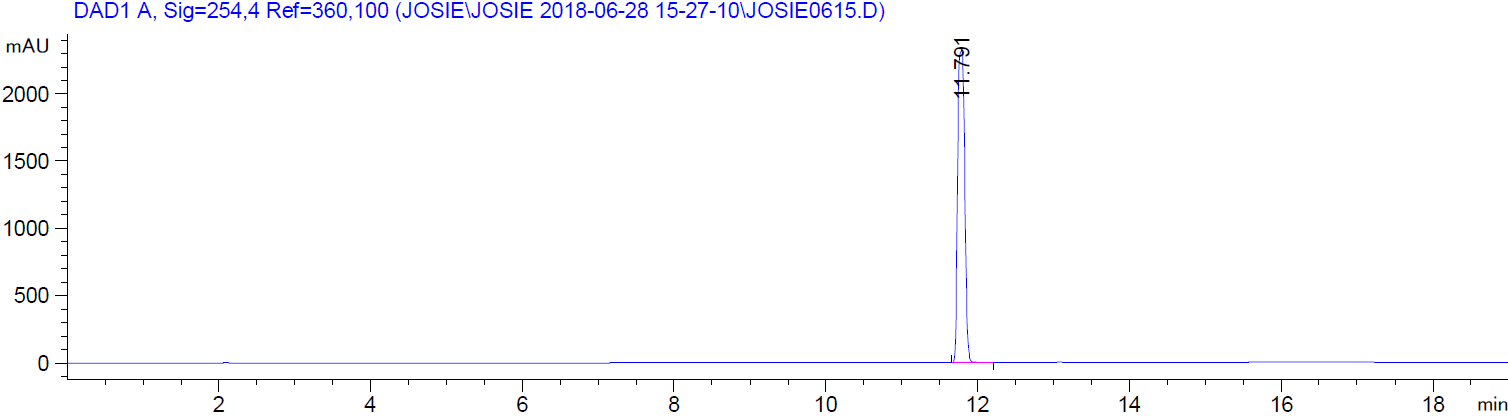


**Figure S4** The HPLC spectra for analysis of pure Flavone using a 5-95% acetonitrile in water gradient.

**NMR comparison of commercially-available flavone and wool fibre**

**
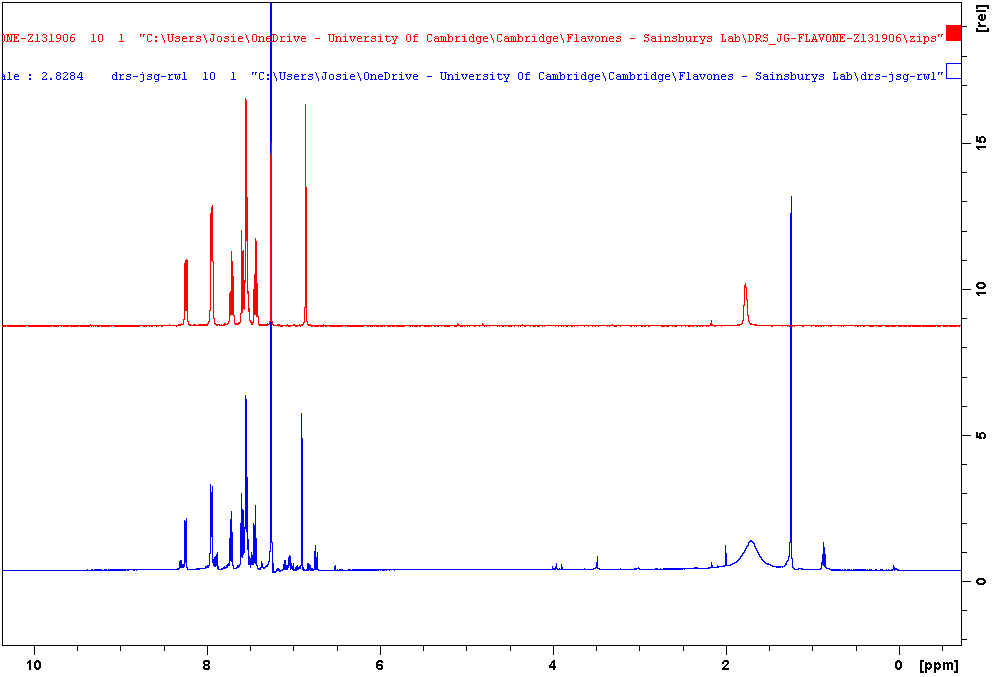
**

**Figure S5a** A comparison of the ^1^H NMR spectra for a pure sample of flavone (red) and the wool fibre sample (blue) between 10 and 0 ppm.

**
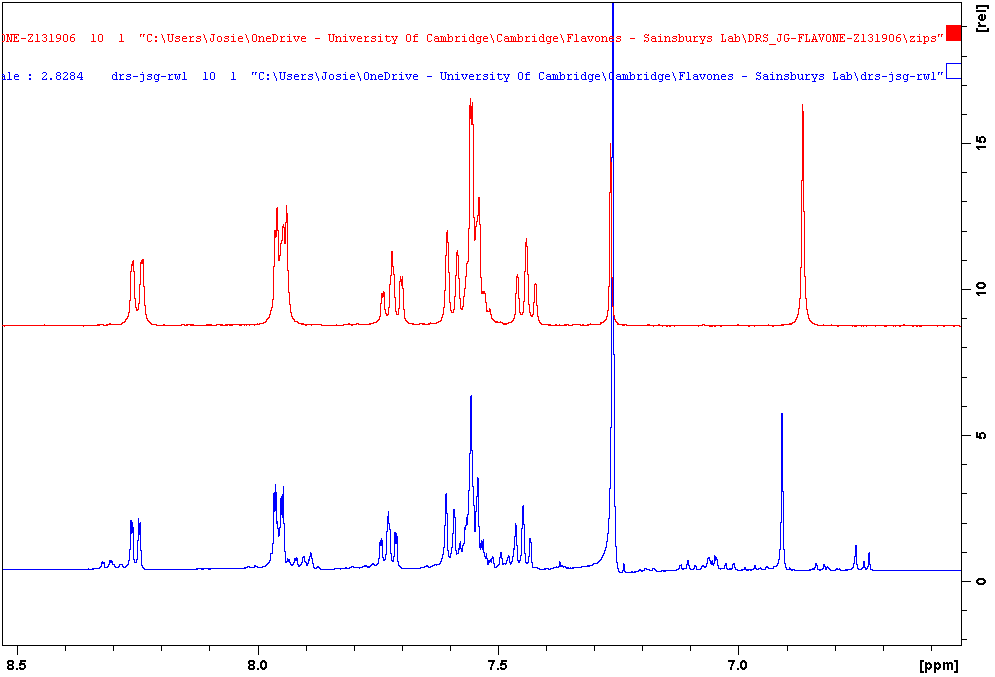
**

**H8**

**H3’**

**H4’**

**H5’**

**H6**

**H7**

**H2’**

**H6’**

**H3**

**H5**

**Figure S5b**A comparison of the ^1^H NMR spectra for a pure sample of flavone (red) and the wool fibre sample (blue) between 8.5 and 6.5 ppm.


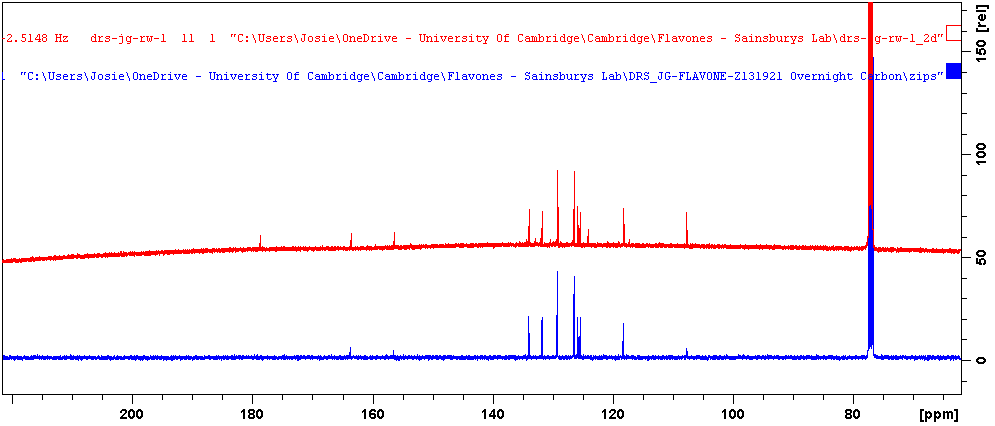


**Figure S5c** A comparison of the ^13^C NMR between 220 and 70 ppm for a pure sample of flavone (red) and the wool fibre sample (blue).

**NMR Analysis of wool fibre sample**

The sample was analysed using the following NMR experiments: ^1^H NMR (Figure S6a), ^13^C NMR (Figure S6b), DEPT 135 (Figure S6c), COSY (Figure S6d), HSQC (Figure S6e) and HMBC (Figure S6f).

The ^1^H and ^13^C NMR of pure flavone were compared to the plant sample and the signals were found to match (S5), providing further evidence that the major component of the plant sample mixture is unsubstituted flavone. The NMR assignments are therefore as follows^[[1]](#footnote-1),^^[[2]](#footnote-2)^:

**^1^H NMR** (500 MHz, CDCl_3_) δ 8.26-8.24 (m, 1H, H5), 7.96-7.94 (m, 2H, H2’, H6’), 7.74-7.71 (m, 1H, H7), 7.60-7.51 (m, 4H, H8, H3’, H4’, H5’), 7.46-7.43 (m, 1H, H6), 6.91 (s, 1H, H3)

**^13^C NMR** (125 MHz, CDCl_3_) δ 178.7 (C, C4), 163.6 (C, C2), 156.5 (C, C9), 134.0 (CH, C7), 131.9 (C, C1’), 131.8 (CH, C3’, C5’), 129.2 (CH, C4’), 126.5 (CH, C2’, C6’), 125.9 (CH, C5), 125.4 (CH, C6), 124.1 (C, C10), 118.3 (CH, C8), 107.8 (CH, C3)




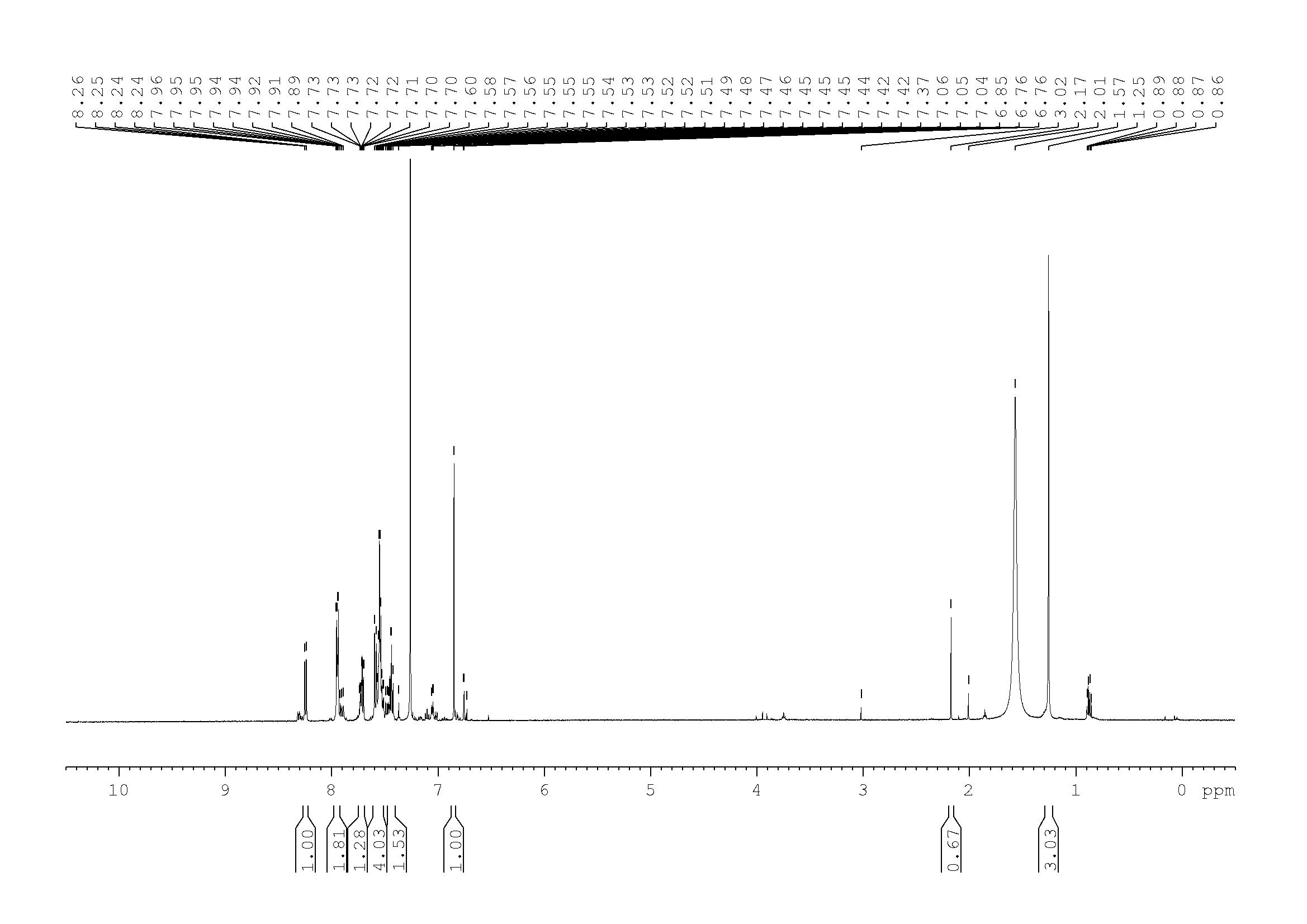


**H3**

**H6**

**H8**

**H3’**

**H4’**

**H5’**

**H2’**

**H6’**

**H7**

**H5**

**Figure S6a** The assigned ^1^H NMR spectrum of the plant sample.




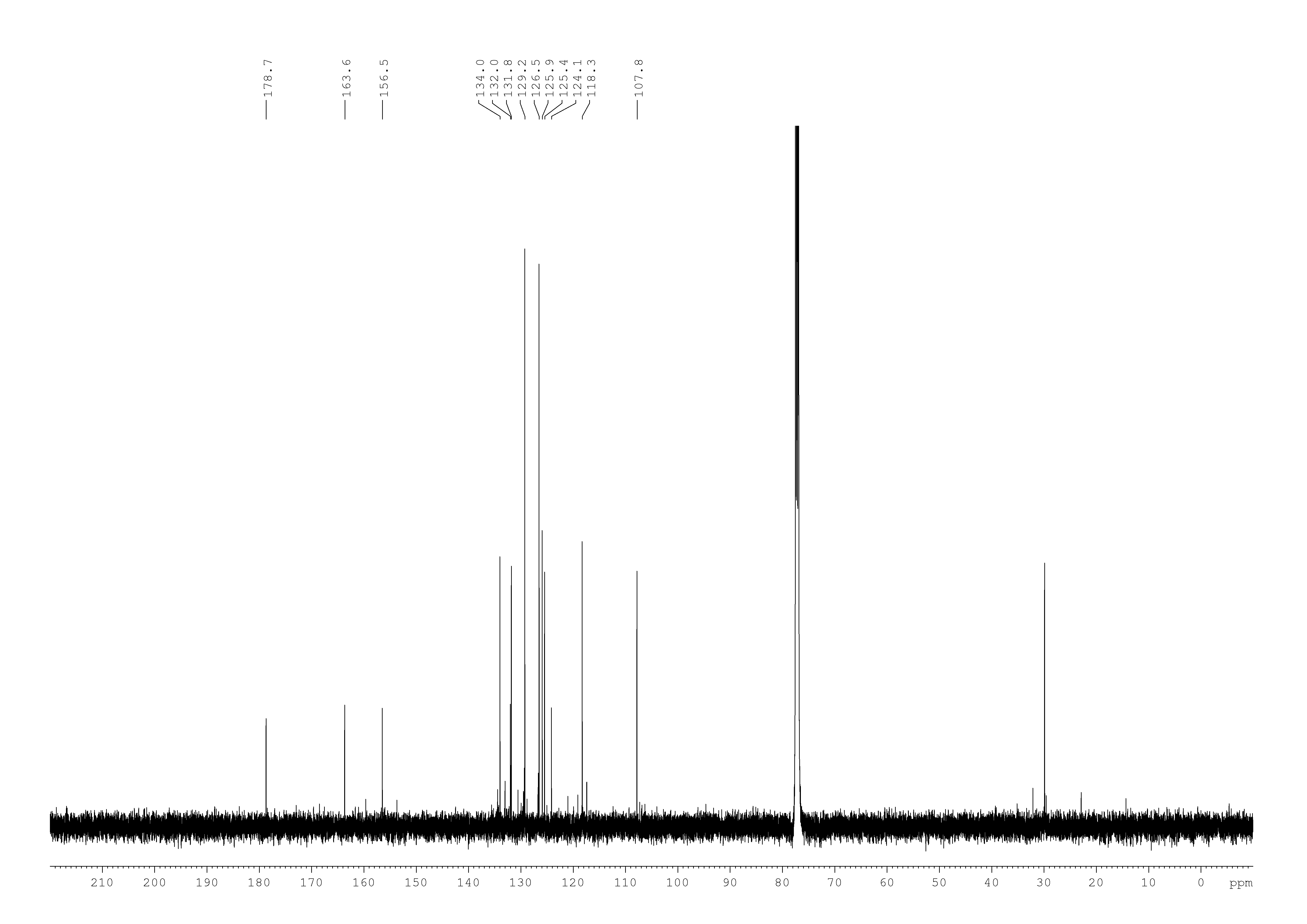


**Figure S6b** The ^13^C NMR spectrum of the plant sample

**
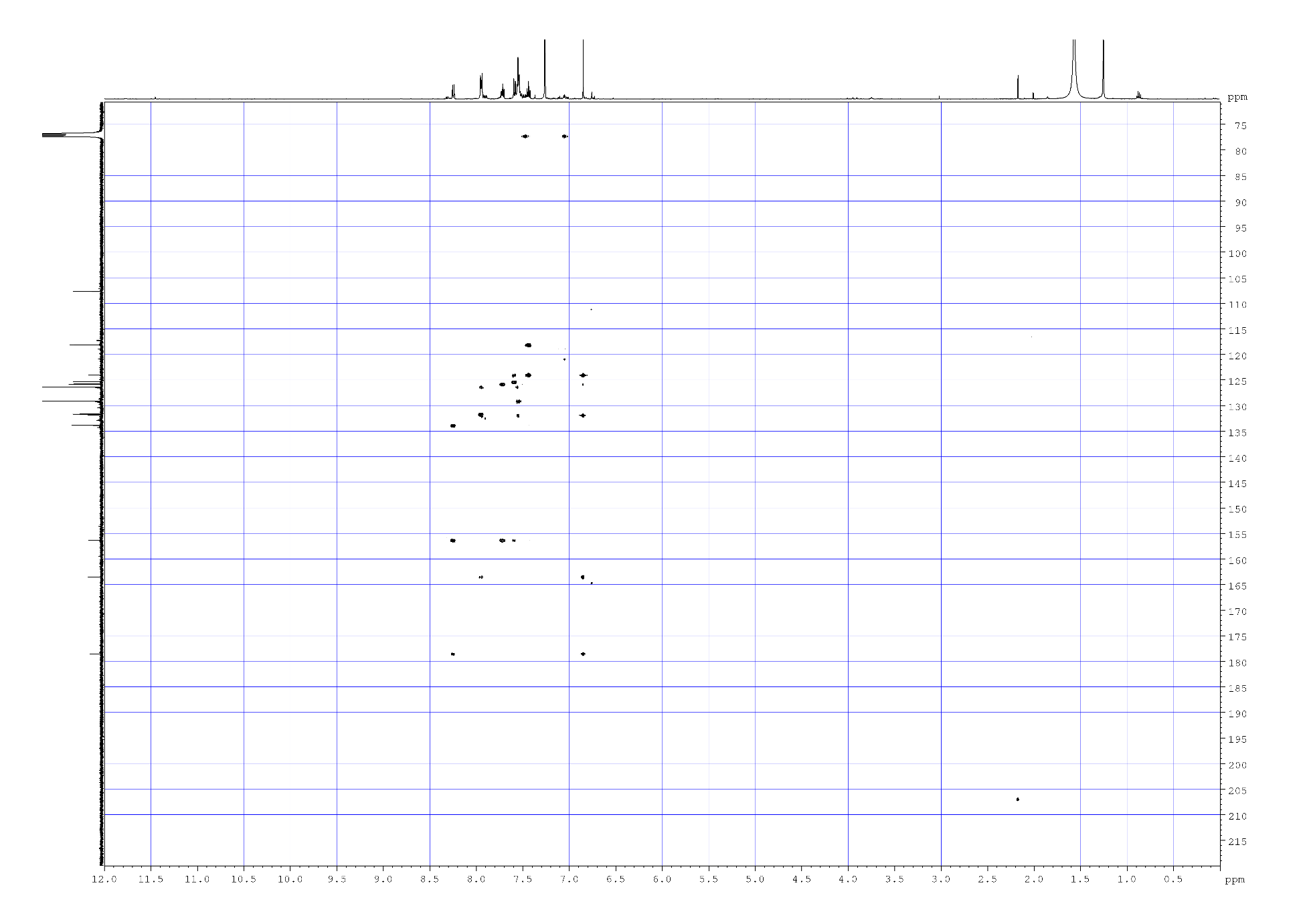
**

**Figure S6c** The HMBC spectrum of the plant sample

**
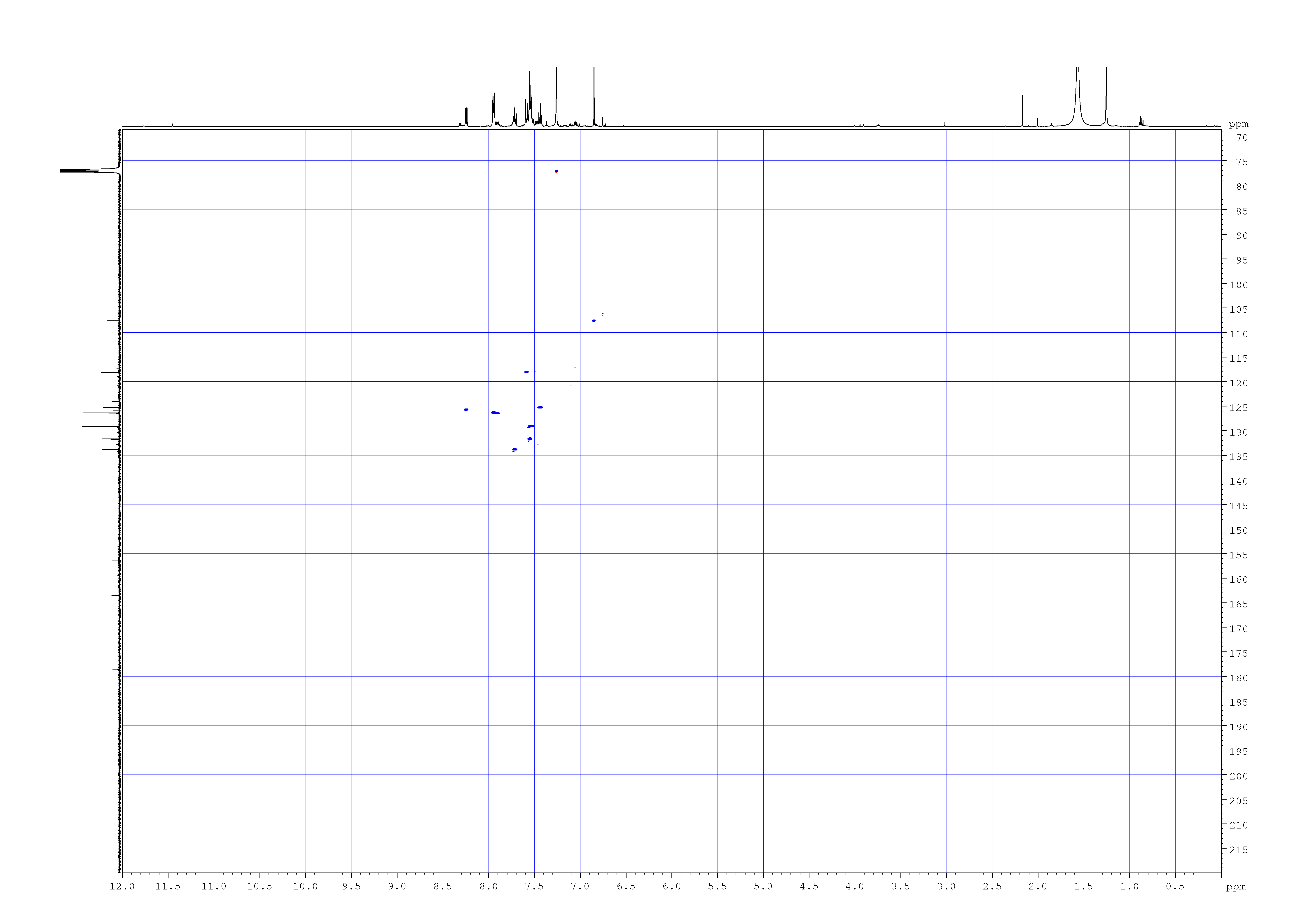
**

**Figure S6d** The HSQC spectrum of the plant sample

**
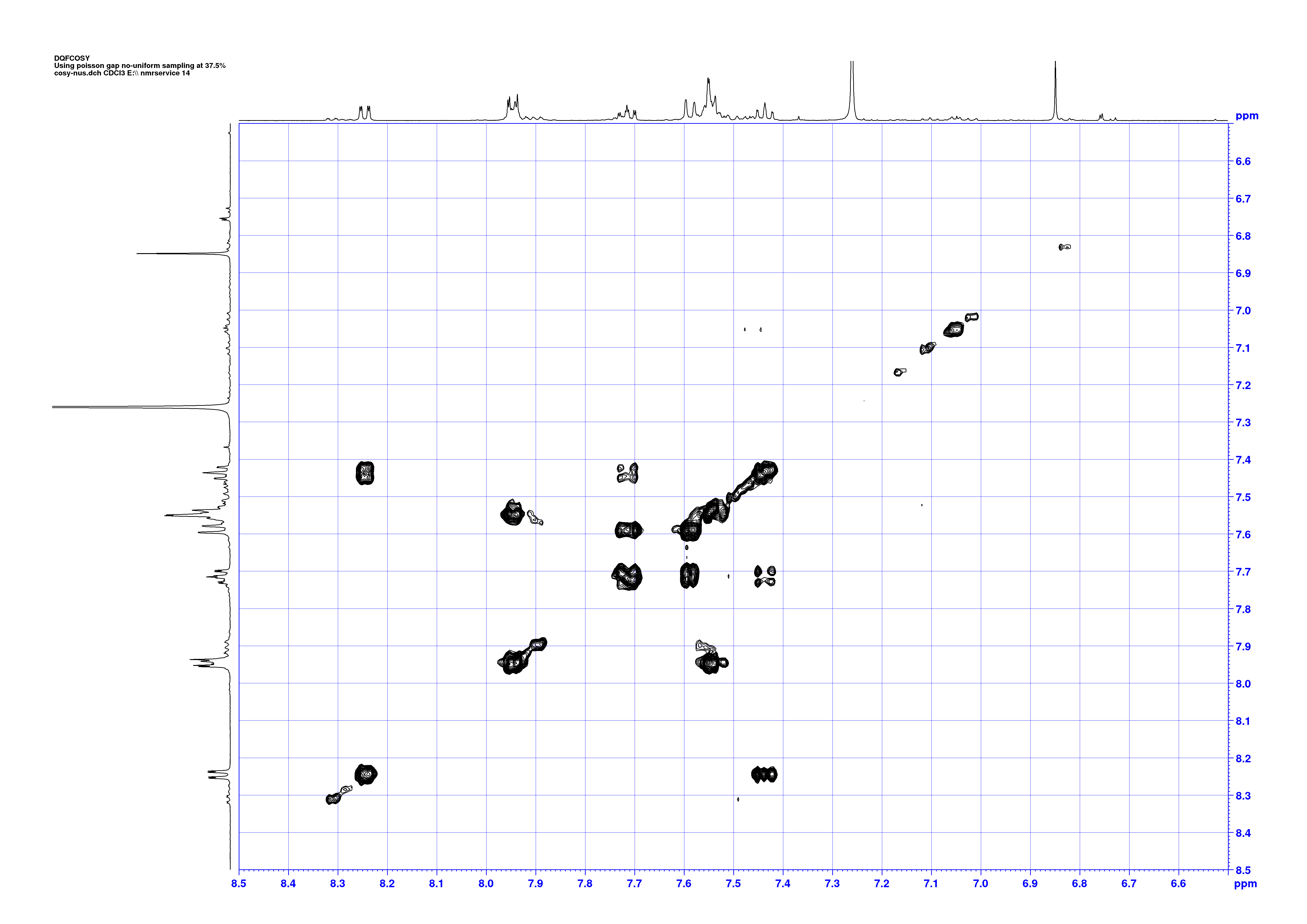
**

**Figure S6e** The COSY spectrum of the plant sample

**
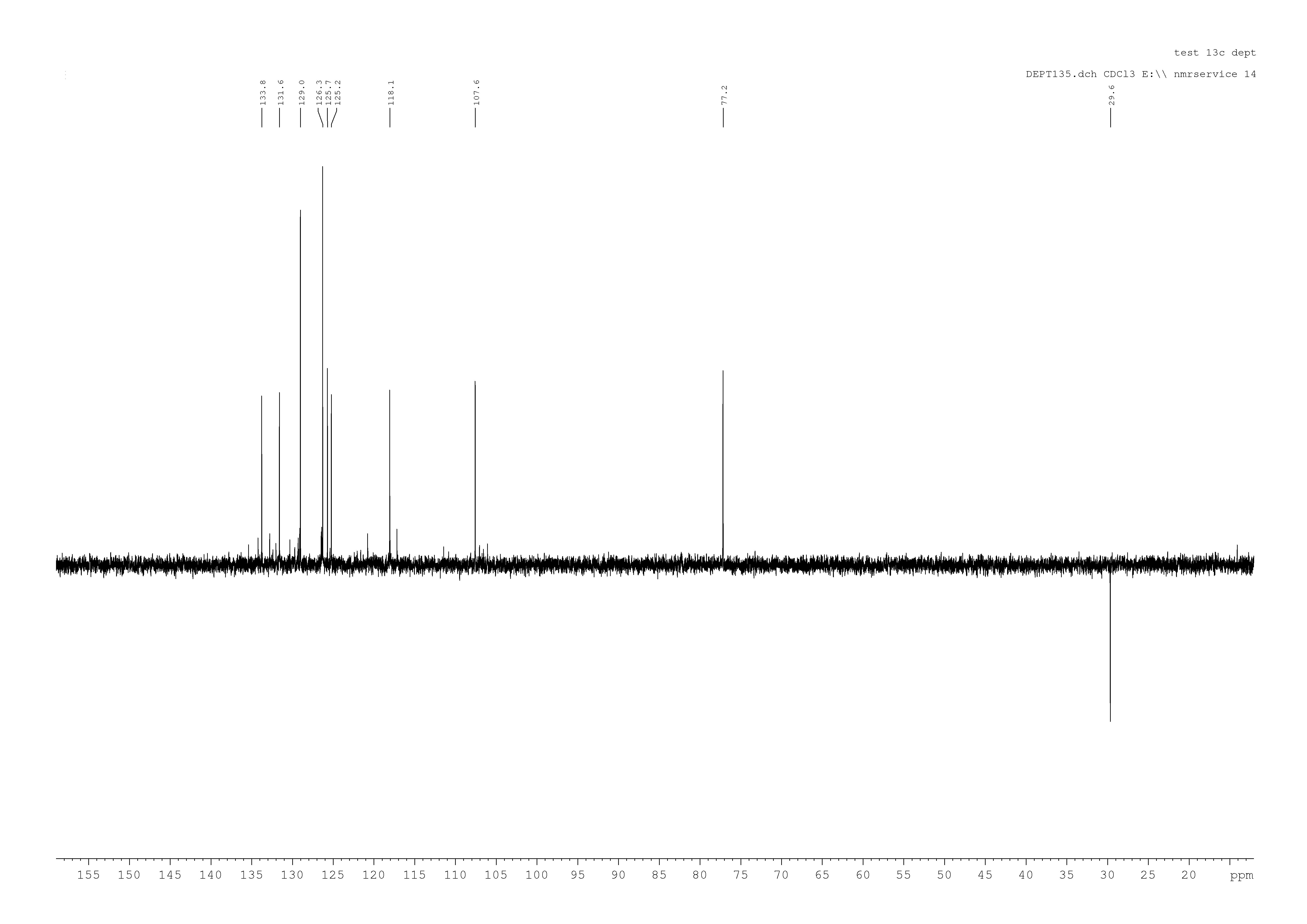
**

**Figure S6f** The DEPT 135 spectrum of the plant sample

**2D NMR experiments to investigate substitution patterns on flavones**

To investigate the structure of the minor species present in the wooly farina sample, a HSQC experiment was run with non-uniform sampling to increase the resolution of the spectrum (Figures S7a and S7b).

**
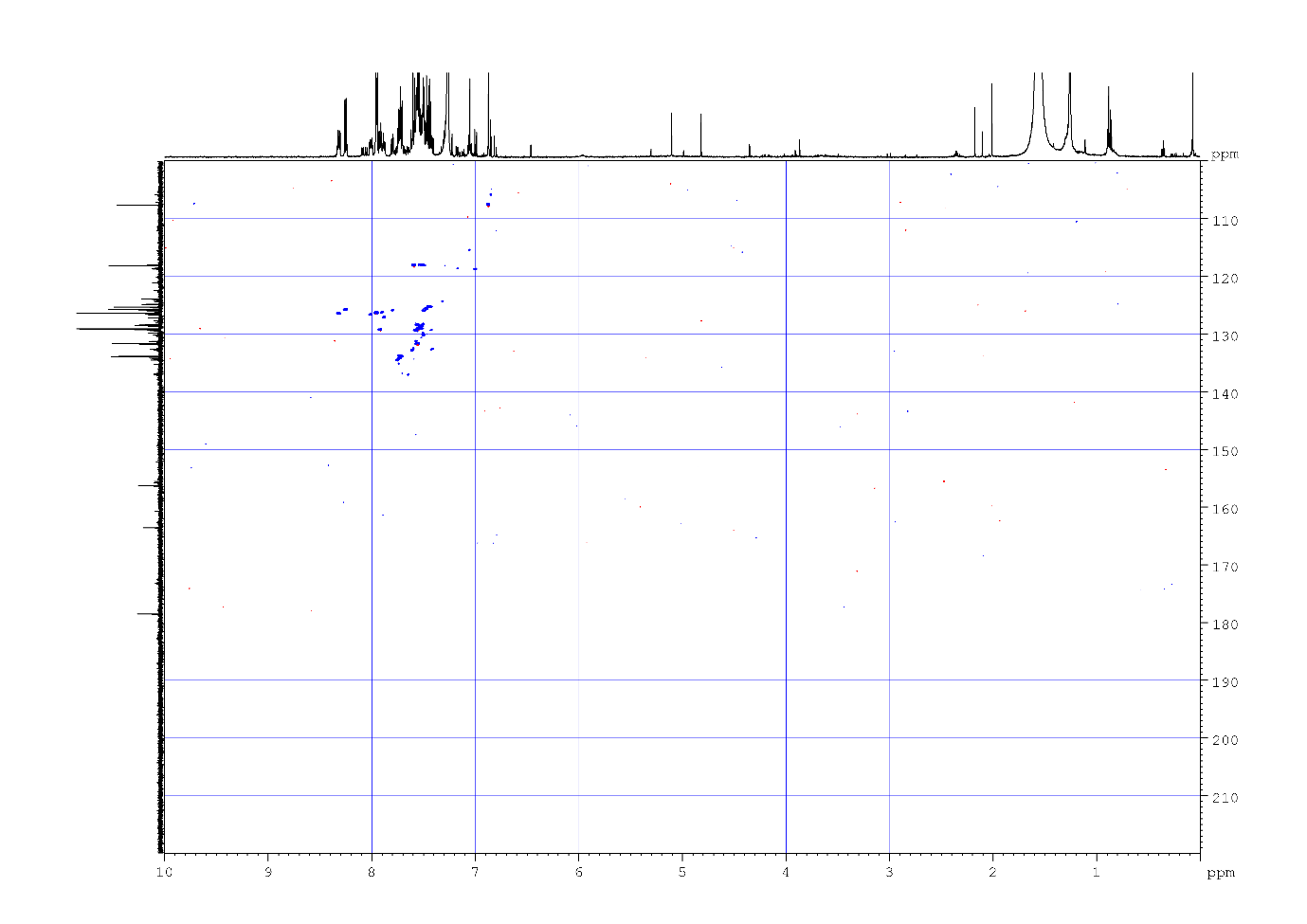
**

**Figure S7a** The spectrum of the HSQC experiment (run with non-uniform sampling).

**
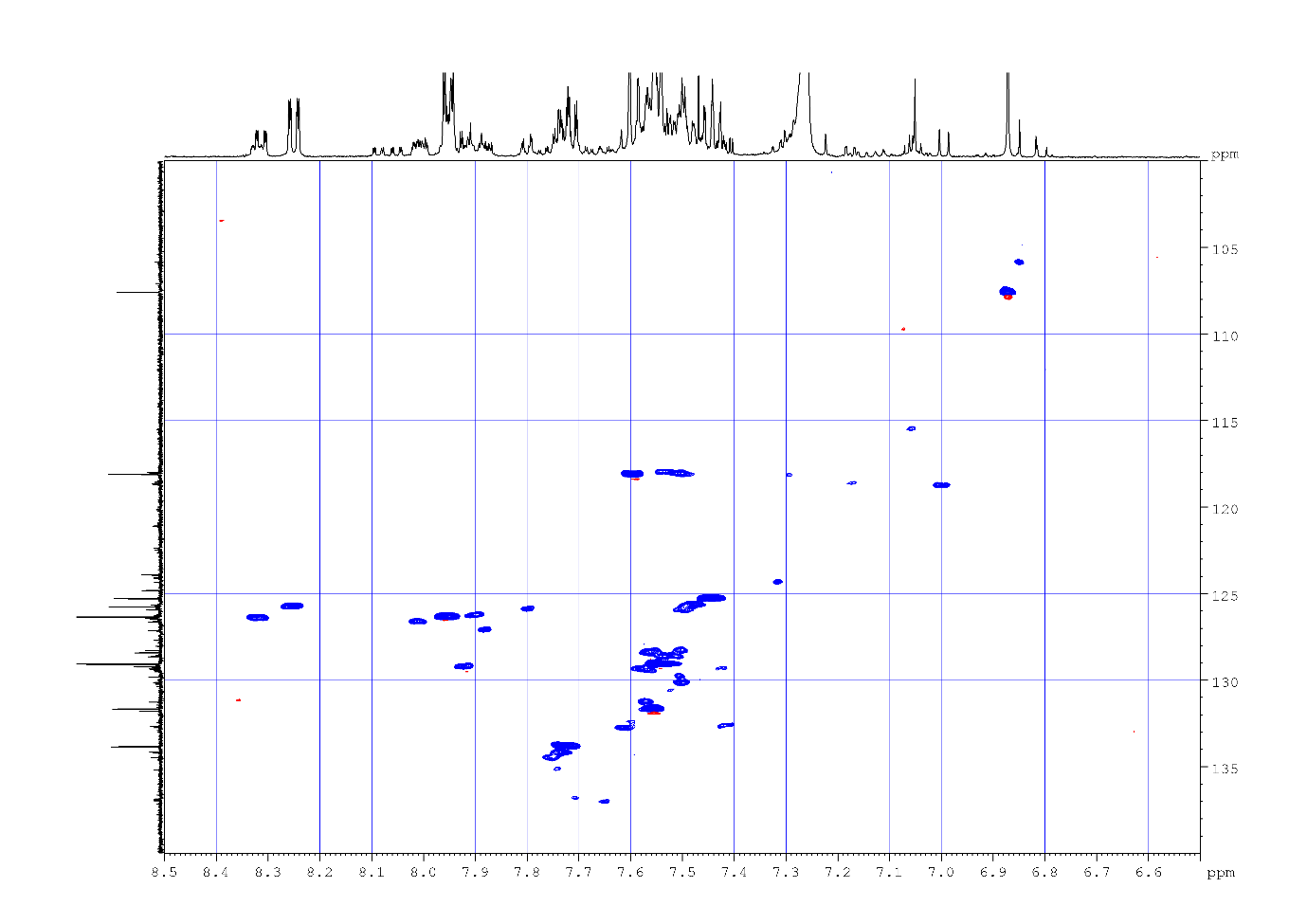
**

**Figure S7b** A zoomed in section of the aromatic region of the HSQC experiment (run with non-uniform sampling).

Without NMR, LCMS, HRMS and HPLC analysis of the isolated components of the plant sample mixture, the structure of the minor species cannot be stated with 100% confidence. However with the data from the LCMS, HPLC, HRMS and NMR, suggestions can be made.

The masses from the LCMS indicated that mono-hydroxy or mono-methoxyflavones were present in the wooly farina sample. Literature values for carbon atoms bearing a hydroxy or methoxy substituent in flavones are very similar and signals are present at 3.9-4.0 ppm in the ^1^H NMR spectrum which could correlate to the methyl group of a methoxy substituent, however with the NMR data available it could not be confirmed if methoxy-substituted flavones were present.

For hydroxy substitution, each potential substitution site was considered in turn. The expected shifts of the protons and carbons with hydroxy-substitution were based on literature values^[[3]](#footnote-3),^^[[4]](#footnote-4)^. From the information provided by the HSQC experiment, the most likely substitution pattern is 4’-hydroxyflavone:

The evidence for this is as follows:

A signal is present in the ^13^C NMR spectrum at 160.7 ppm which is in the correct region for the quaternary C4’ carbon with either a hydroxyl substitution (Figure S7c).

Correlation is observed in the HSQC between a ^13^C signal at 127.1 ppm and a ^1^H signal at 7.88 ppm (A, Figure S7d). These match the expected shifts for an aromatic CH in a meta position to the hydroxylation site (i.e. the CH at either C2’ or C6’).

Correlation is observed between a ^13^C signal at 118.6 ppm and a ^1^H signal at 7.16 ppm (B, Figure S7d) which match the expected shifts for an aromatic CH in an ortho position relative to the hydroxyl group (i.e. the CH at C3’ or C5’).


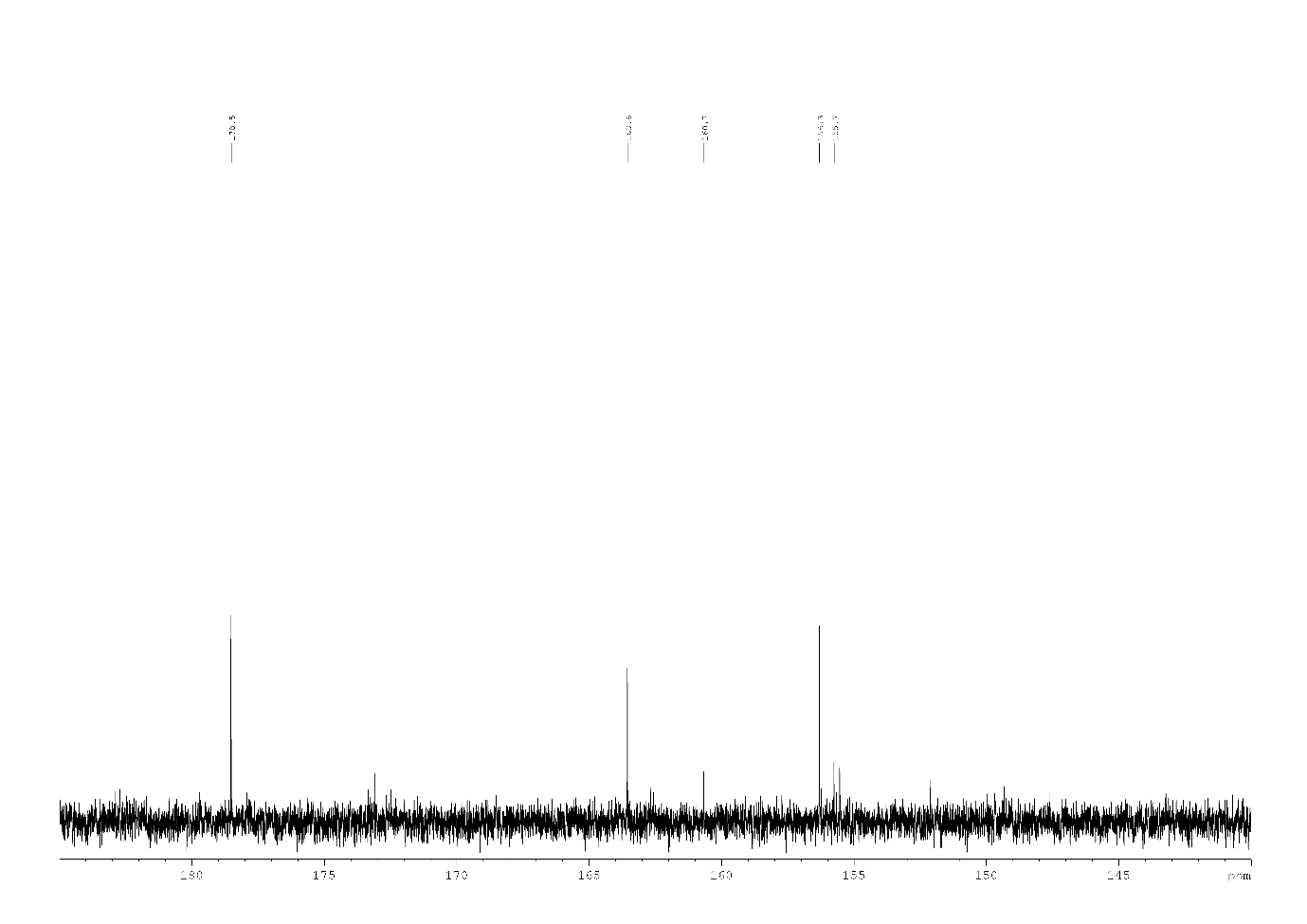


**Figure S7c** The ^13^C NMR spectrum showing the peak at 160.3 ppm


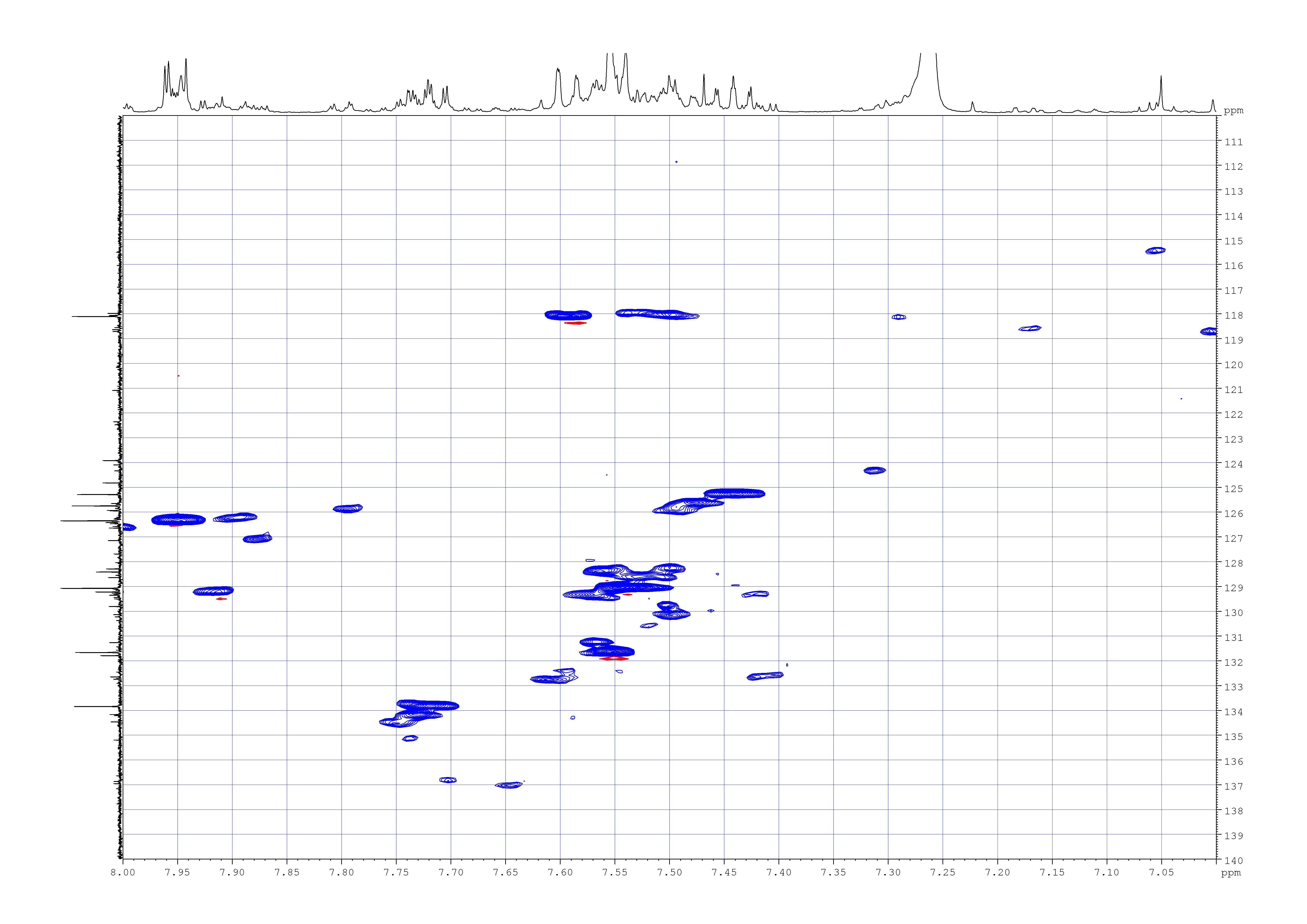


**B**

**A**

**Figure S7d** A zoomed in region of the HSQC (with non-uniform sampling) showing the two correlations A and B.

As the LCMS data indicated there were two mono-hydroxylated species with different retention times present in the plant sample, there could be another isomer present. The NMR data point towards this being 2’-hydroxyflavone:

The evidence for this includes:

In the ^13^C NMR spectrum there is a peak at 152.1 ppm, which is around the expected shift for the quaternary C2’ bearing the hydroxyl group (Figure S7e).

In the HSQC experiment with non-uniform sampling, a peak at 132.6 ppm in the ^13^C NMR correlates with a peak at 7.41 ppm in the ^1^H NMR (A, Figure S7f). These shifts match the expected signal for an aromatic CH which is in a meta position relative to the hydroxyl (i.e. the signal for H4’)

In the HSQC experiment with non-uniform sampling, a peak at 124.3 ppm in the ^13^C NMR correlates with a peak at 7.31 ppm in the ^1^H NMR (B, Figure S7f). These shifts match the expected signal for an aromatic CH which is in a para position to the hydroxyl group (i.e. for C5’).


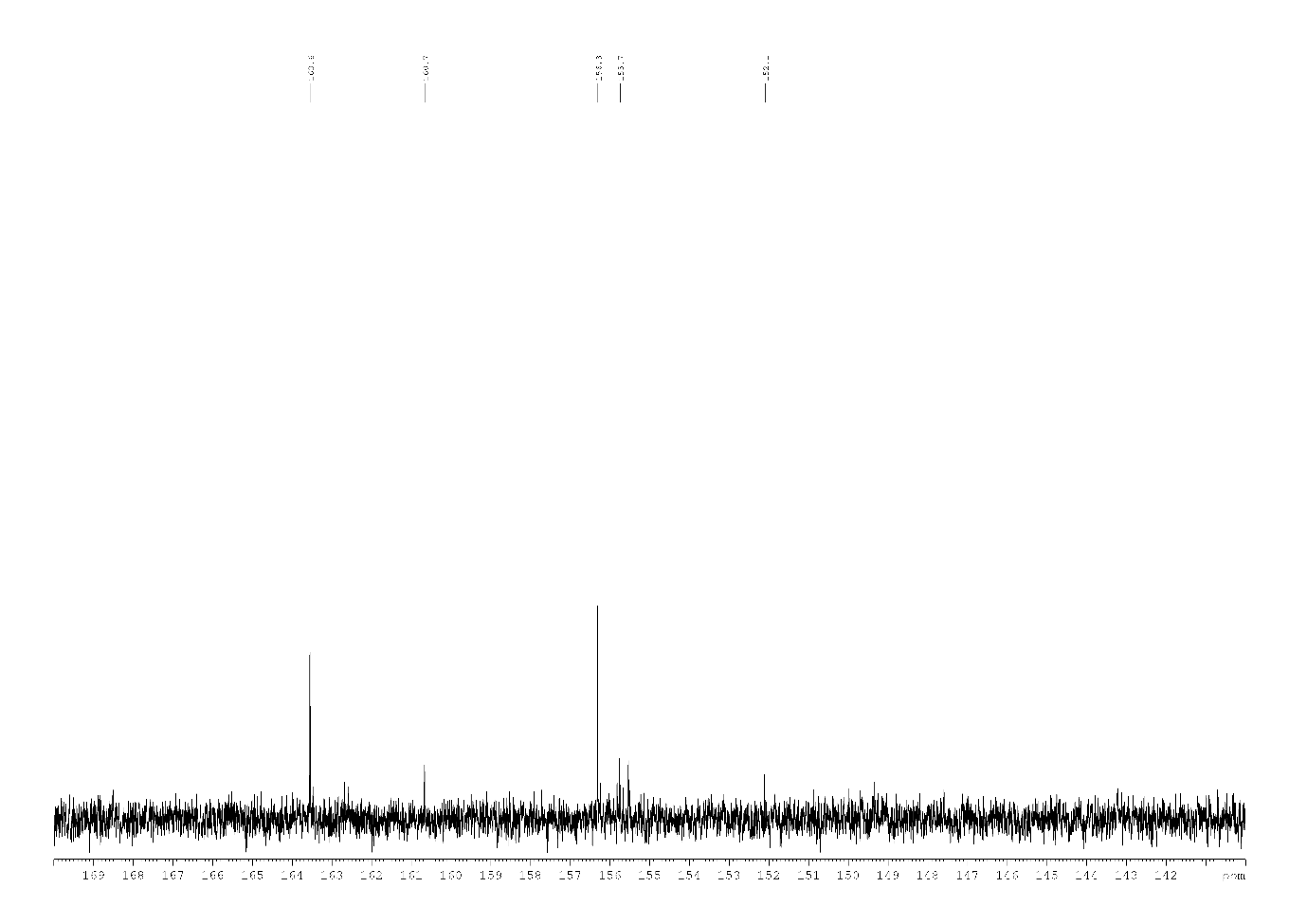


**Figure S7e** A zoomed in region of the HSQC (with non-uniform sampling) showing the two correlations A and B.


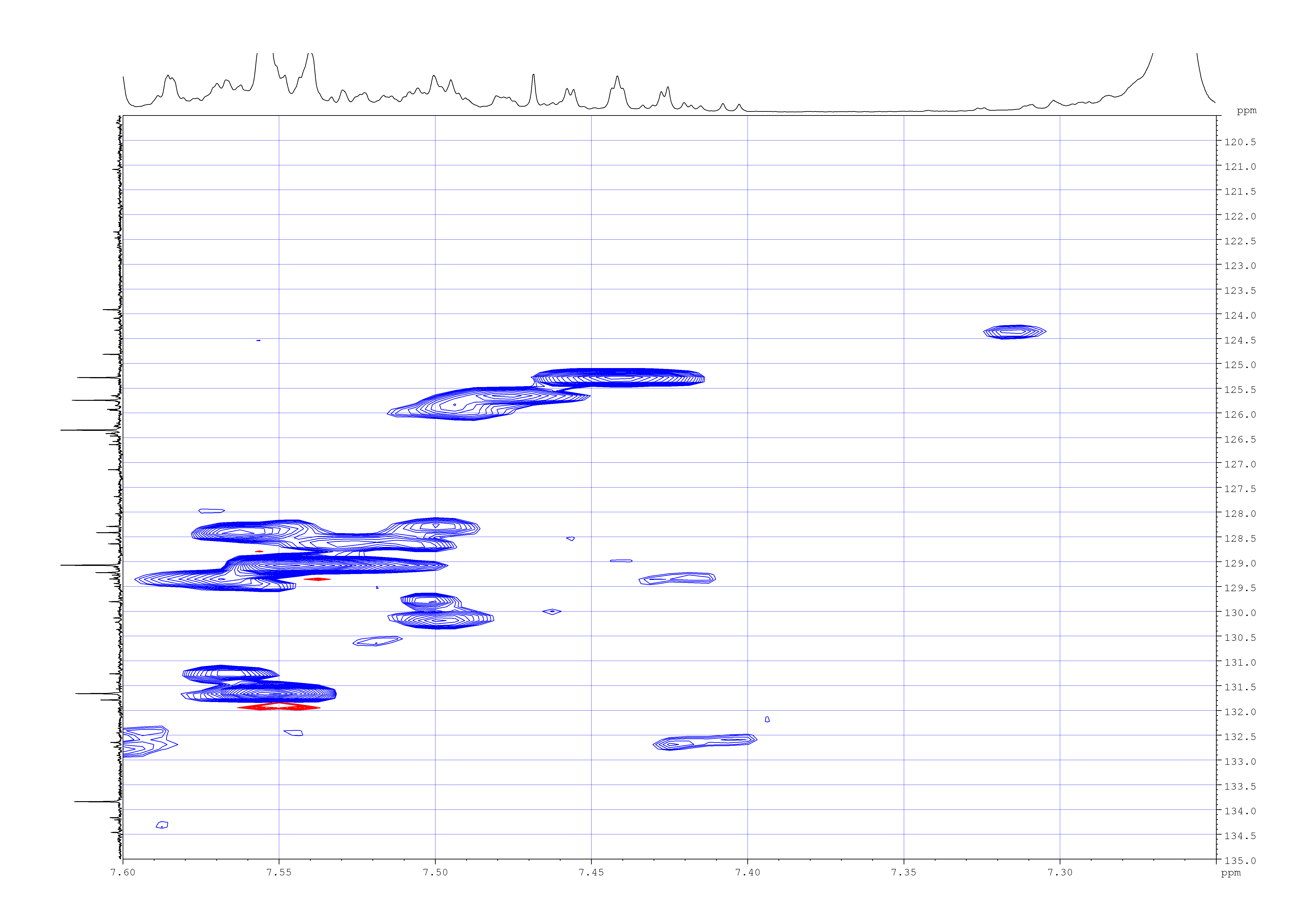


**A**

**B**

**Figure S7f** A zoomed in region of the HSQC (with non-uniform sampling) showing the two correlations A and B.

1. B.-H. Moon, Y. Lee, C. Shin, Y. Lim, Complete Assignments of the ^1^H and ^13^C NMR Data of Flavone Derivatives, *Bull. Korean Chem. Soc.* **26** (4), 603-608 (2005) [↑](#footnote-ref-1)
2. # D. W. Aksnes, A. Standnes, Ø. M. Andersen, Complete Assignment of the ^1^H and ^13^C NMR Spectra of Flavone and its A‐Ring Hydroxyl Derivatives, *Magnetic Resonance in Chemistry* 34, 820-823 (1996)

   [↑](#footnote-ref-2)
3. D. H. Williams, I. Flemming, *Spectroscopic Methods in Organic Chemistry* (McGraw-Hill Companies, ed. 5, 1996) [↑](#footnote-ref-3)
4. Y. Park, B.-H. Moon, E. Lee, Y. Lee, Y. Yoon, J.-H. Ahn, Y. Lim, *Magn. Reson. Chem*. **45**, 674–679 (2007) [↑](#footnote-ref-4)
