# Supplementary material for: Microscopy and chemical analyses reveal flavone-based woolly fibres extrude from micron-sized holes in glandular trichomes of Dionysia tapetodes
Source: BMC Plant Biol. 2021 Jun 17;21:258. doi: 10.1186/s12870-021-03010-9 (PMC8210372; doi:10.1186/s12870-021-03010-9)
Supplement: Supplementary file 3 — Additional file 3. Confocal transmitted image (A, C) and cell wall fluorescence (B, D) of calcofluor-stained sections through gland hair cells. Wool exit holes, observed as discrete gaps in the fluorescence images (arrows) are in close proximity to the dense vacuole (V). [file 12870_2021_3010_MOESM3_ESM.pdf]

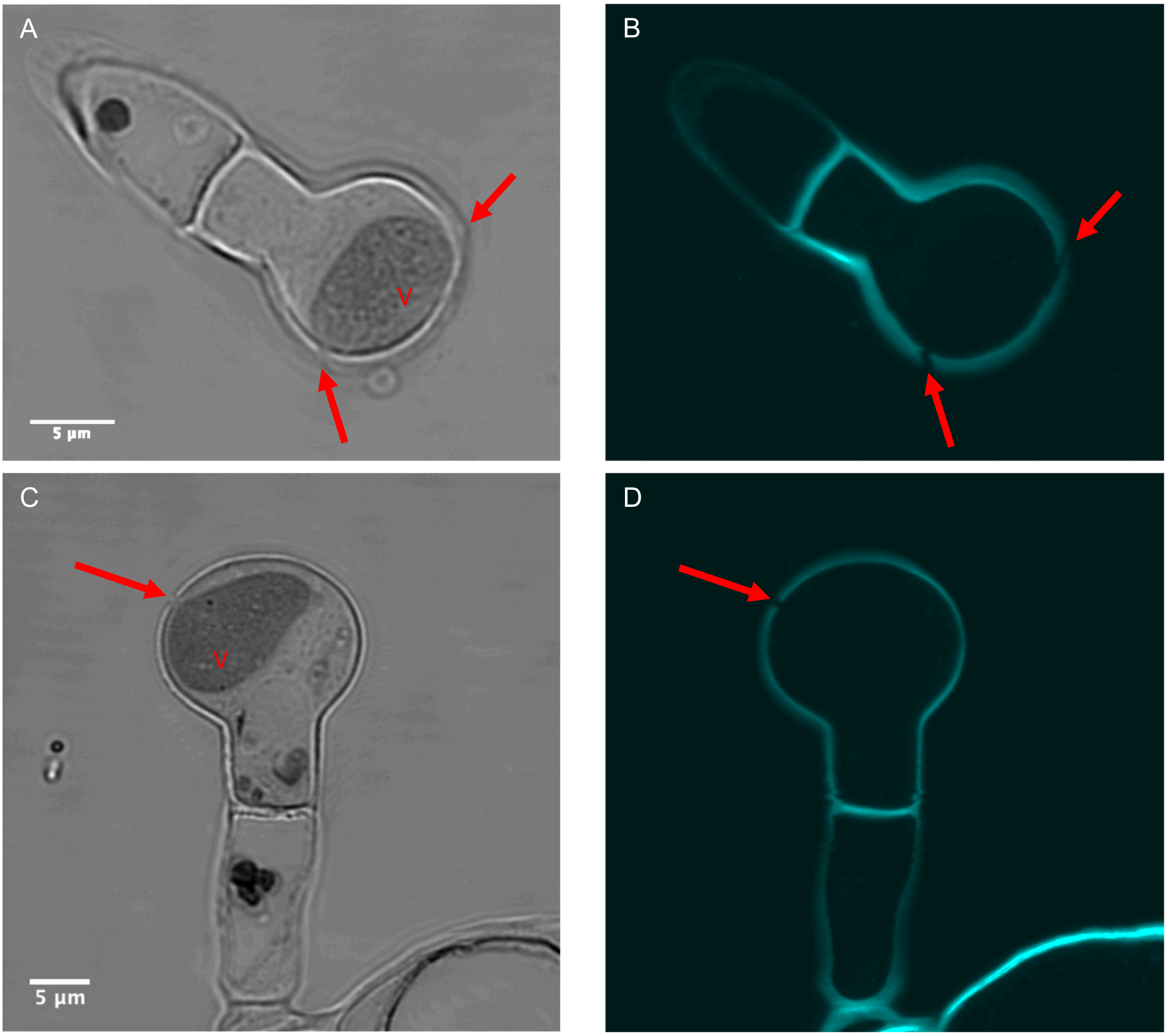

Additional file 3. Confocal transmitted image (A, C) and cell wall fluorescence (B, D) of calcofluor-stained sections through gland hair cells. Wool exit holes, observed as discrete gaps in the fluorescence images (arrows) are in close proximity to the dense vacuole (V).
