# Supplementary material for: Microscopy and chemical analyses reveal flavone-based woolly fibres extrude from micron-sized holes in glandular trichomes of Dionysia tapetodes
Source: BMC Plant Biol. 2021 Jun 17;21:258. doi: 10.1186/s12870-021-03010-9 (PMC8210372; doi:10.1186/s12870-021-03010-9)
Supplement: Supplementary file 4 — Additional file 4. Fluorescence Lifetime Imaging (FLIM) of glandular trichomes taken together with Raman spectra acquired along a line through a trichome cell. The FLIM data (centre left image) represents lifetimes of autofluorescence. Farinose material including the wool and edge of the trichome cell have short lifetimes (cyan and blue colours) with high signal and the cell interior has blue (short) and green (long) lifetimes with low signal. Raman measurements at precise locations along the line confirm the presence of flavone-type material at the cell edge together with strong peaks equivalent to those of plant epicuticular wax (Upper spectrum, assignments are given for prominent peaks). Note the intense cyan labelling in the FLIM image that is a similar lifetime to the woolly farina (white boxed region). Within a proximal location inside the cell there is an absence of the wax-associated peaks and the strong flavone peaks (lower spectrum). Carotenoid is detected at both locations. Another location inside the cell yielded strong flavone peaks (centre spectrum) and may represent an intracellular store of flavones. [file 12870_2021_3010_MOESM4_ESM.pdf]

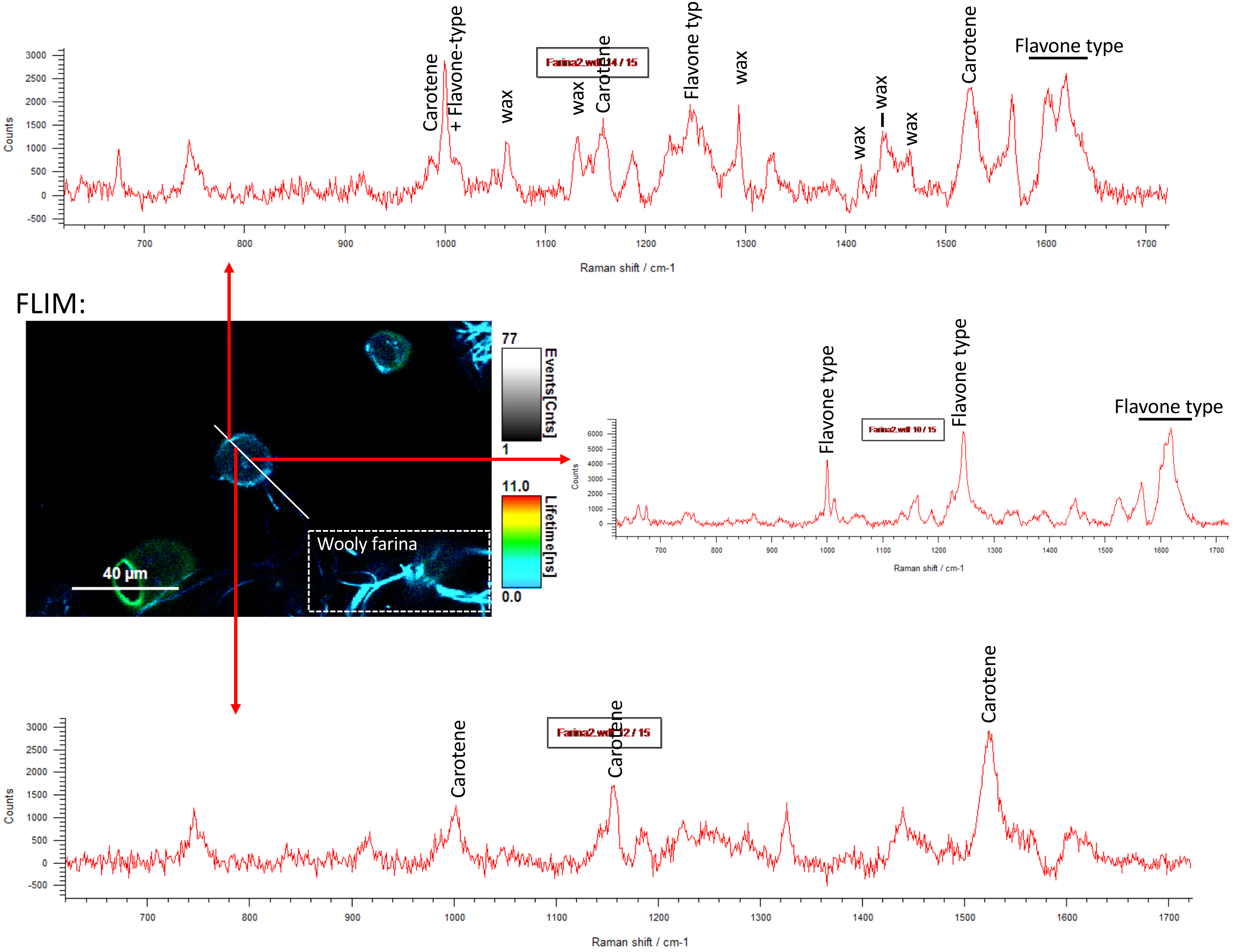
Carotene
+ Flavone-type
wax
wax
Carotene
Flavone typ
wax
wax
wax
wax
Carotene
Flavone type
Farina2.wdf 14 / 15
Counts
Raman shift / cm-1
FLIM:
77
Events[Cnts]
1
11.0
Lifetime[ns]
0.0
40 µm
Wooly farina
Flavone type
Flavone type
Flavone type
Farina2.wdf 10 / 15
Counts
Raman shift / cm-1
Carotene
Carotene
Carotene
Farina2.wdf 12 / 15
Counts
Raman shift / cm-1

Additional file 4. Fluorescence Lifetime Imaging (FLIM) of glandular trichomes taken together with Raman spectra acquired along a line through a trichome cell. The FLIM data (centre left image) represents lifetimes of autofluorescence. Farinose material including the wool and edge of the trichome cell have short lifetimes (cyan and blue colours) with high signal and the cell interior has blue (short) and green (long) lifetimes with low signal. Raman measurements at precise locations along the line confirm the presence of flavone-type material at the cell edge together with strong peaks equivalent to those of plant epicuticular wax (Upper spectrum, assignments are given for prominent peaks). Note the intense cyan labelling in the FLIM image that is a similar lifetime to the woolly farina (white boxed region). Within a proximal location inside the cell there is an absence of the wax-associated peaks and the strong flavone peaks (lower spectrum). Carotenoid is detected at both locations. Another location inside the cell yielded strong flavone peaks (centre spectrum) and may represent an intracellular store of flavones.
