# Supplementary material for: Microscopy and chemical analyses reveal flavone-based woolly fibres extrude from micron-sized holes in glandular trichomes of Dionysia tapetodes
Source: BMC Plant Biol. 2021 Jun 17;21:258. doi: 10.1186/s12870-021-03010-9 (PMC8210372; doi:10.1186/s12870-021-03010-9)
Supplement: Supplementary file 5 — Additional file 5. FE-SEM images showing intact and continuous membranes from young trichomes and leaf cells. Note these images were taken from the same section as the farina producing glandular trichomes shown in Fig. 4j-n. [file 12870_2021_3010_MOESM5_ESM.pdf]

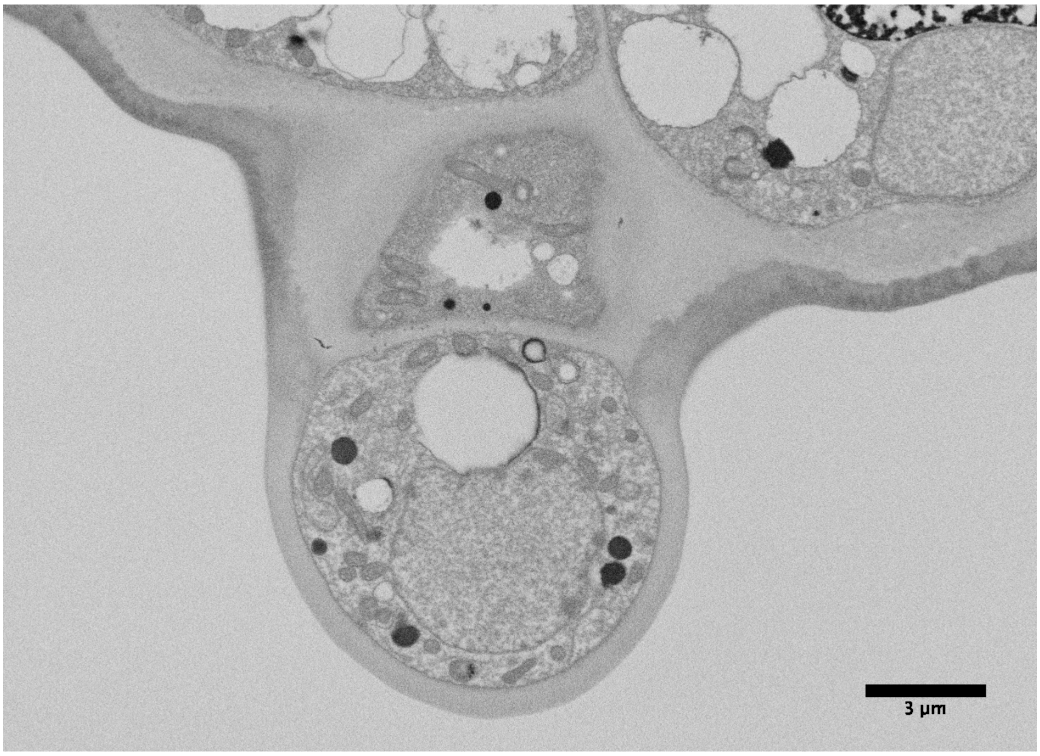

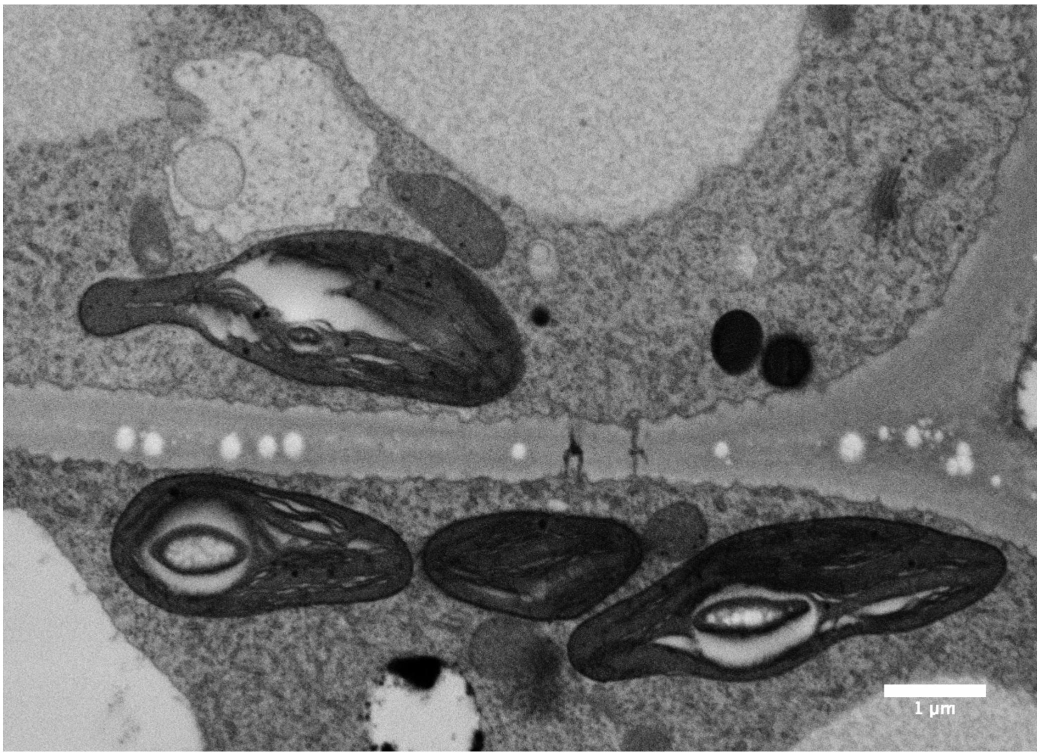

Additional file 5. FE-SEM images showing intact and continuous membranes from young trichomes and leaf cells. Note these images were taken from the same section as the farina producing glandular trichomes shown in Fig. 4. J-N.
