# Supplementary material for: Microscopy and chemical analyses reveal flavone-based woolly fibres extrude from micron-sized holes in glandular trichomes of Dionysia tapetodes
Source: BMC Plant Biol. 2021 Jun 17;21:258. doi: 10.1186/s12870-021-03010-9 (PMC8210372; doi:10.1186/s12870-021-03010-9)
Supplement: Supplementary file 6 — Additional file 6. FE-SEM large area tile-scan of a section thorough the rosette leaves of D. tapetodes. The high resolution tile images enable organelles to be resolved. Magnified examples of glandular head cells and nearby leaf mesophyll cells are shown. Red arrows identify candidate electron-transparent organelles that may be lipid droplets. Yellow arrows identify similar sized electron dense droplets. The full resolution tiled image of size 35,172 x 46,156 pixels is deposited at http://dx.doi.org/10.17632/tk534bkb85.1. [file 12870_2021_3010_MOESM6_ESM.pdf]

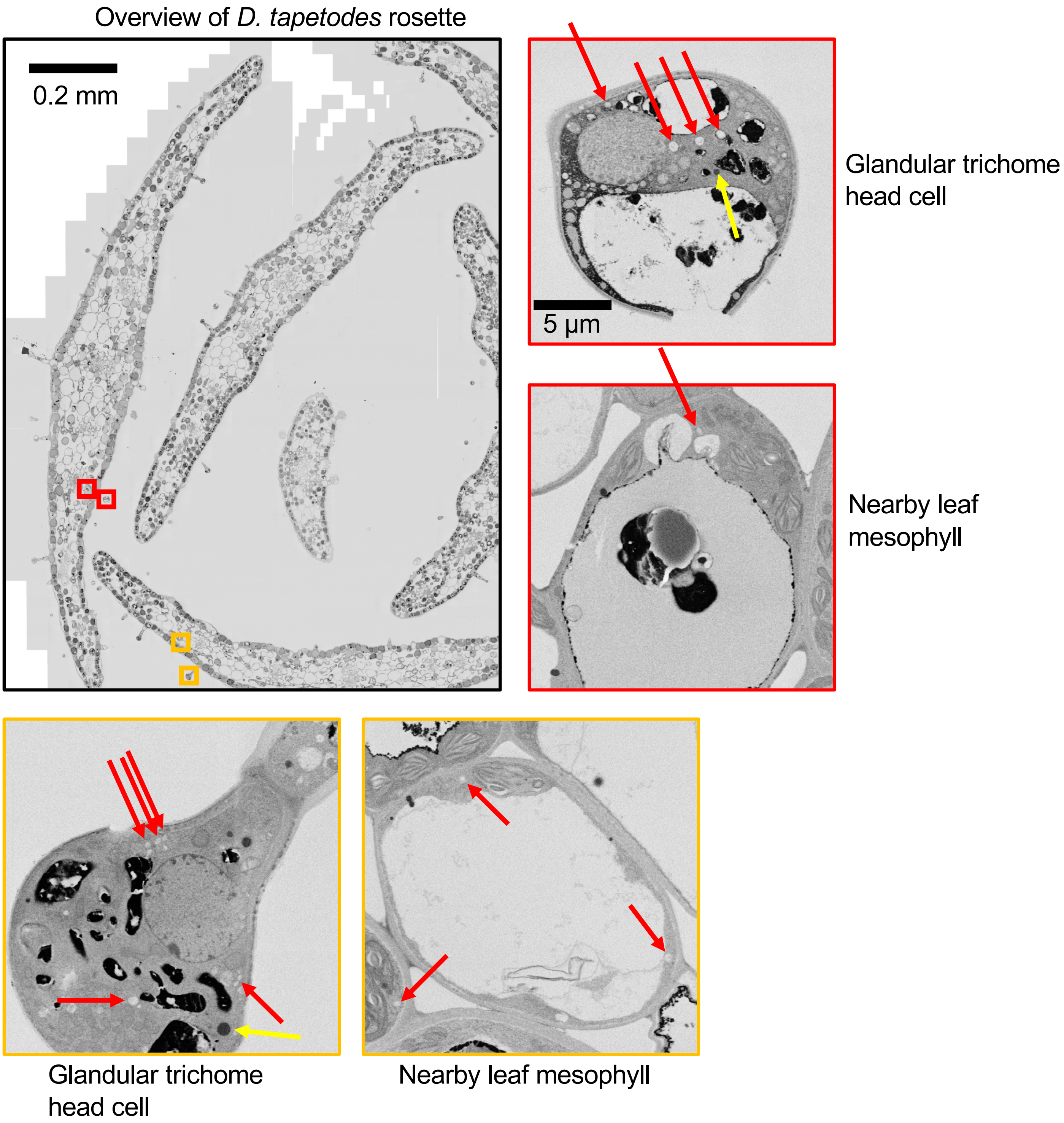

Additional file 6. FE-SEM large area tile-scan of a section thorough the rosette leaves of *D. tapetodes*. The high resolution tile images enable organelles to be resolved. Magnified examples of glandular head cells and nearby leaf mesophyll cells are shown. Red arrows identify candidate electron-transparent organelles that may be lipid droplets. Yellow arrows identify similar sized electron dense droplets. The full resolution tiled image of size 35,172 x 46,156 pixels, enabling detailed observations of all cells, is deposited at http://dx.doi.org/10.17632/tk534bkb85.1.
